# Gross Antioxidant Capacity and Anti-Inflammatory Potential of Flavonol Oxidation Products: A Combined Experimental and Theoretical Study

**DOI:** 10.3390/antiox14040479

**Published:** 2025-04-16

**Authors:** Karen Acosta-Quiroga, Esteban Rocha-Valderrama, Matías Zúñiga-Bustos, Raúl Mera-Adasme, Gustavo Cabrera-Barjas, Claudio Olea-Azar, Mauricio Moncada-Basualto

**Affiliations:** 1 Laboratorio de Radicales Libres y Antioxidantes, Facultad de Ciencias Químicas y Farmacéuticas, Universidad de Chile, Santiago 8380494, Chile; erocha@utem.cl (E.R.-V.); colea@uchile.cl (C.O.-A.); 2 Instituto Universitario de Investigación y Desarrollo Tecnológico, Universidad Tecnológica Metropolitana, Santiago 8940577, Chile; mzunigab@utem.cl; 3 Departamento de Química, Facultad de Ciencias, Universidad de Tarapacá, Arica 1000007, Chile; rmeraa@academicos.uta.cl; 4 Facultad de Ciencias de la Rehabilitación y Calidad de Vida, Universidad San Sebastián, Campus Las Tres Pascualas, Lientur 1457, Concepción 4080871, Chile; gustavo.cabrera@uss.cl

**Keywords:** flavonol oxidation, benzofuranones, antioxidant capacity, ORAC assays, DFT analysis, molecular docking and COX-2 inhibition

## Abstract

This study evaluated the antioxidant capacity of the oxidation products of three flavonols using oxygen radical absorbance capacity—fluorescein assay (ORAC-FL), oxygen radical absorbance capacity—pyrogallol red assay (ORAC-PGR), and the cellular antioxidant activity (CAA) assay in human dermal fibroblast (HFF) cells, with 2,2’-azobis(2-amidinopropane) dihydrochloride (AAPH) as a free radical generator under controlled pH and solvent conditions. At pH 2 in a polar aprotic solvent, BZF-OH (benzofuranone-OH) compounds were formed, while methoxylated analogs were obtained at pH 7 in a polar protic solvent. The products generated at pH 2 exhibited significantly higher antioxidant capacities, demonstrating the influence of the reaction environment on modulating antioxidant properties. The antioxidant activity was observed to reflect the combined action of the flavonol precursor and its oxidation products. This led to the proposal of the Gross Antioxidant Capacity (GAC) concept to integrate the contribution of all generated species. Since chemical assays such as ORAC do not fully capture the complexity of biological systems, they should be complemented with cellular approaches for a more accurate evaluation. Additionally, BZF-OH compounds were analyzed as potential cyclooxygenase-2 (COX-2) inhibitors through docking and molecular dynamics simulations, where BZF-Quer-OH showed binding affinities comparable to celecoxib, a selective COX-2 inhibitor. These findings were complemented by an analysis of COX-2 expression in RAW 264.7 cells treated with lipopolysaccharide (LPS), where treatment with the antioxidants significantly inhibited COX-2 expression. In the case of the oxidation products, only the oxidation product of rhamnetin showed a reduction in COX-2 expression compared to the LPS-treated control. Together, these results highlight that flavonol-derived oxidation products not only retain significant antioxidant capacity but may also possess anti-inflammatory properties, opening new perspectives for the development of innovative therapies targeting oxidative stress and chronic inflammation.

## 1. Introduction

Reactive oxygen species (ROS) and reactive nitrogen species (RNS) are molecules that react oxidatively with various biological targets [1], playing a crucial role in oxidative stress [2,3]. This stress results from an imbalance between the production of oxidants and the body’s ability to neutralize them through antioxidants; these reactive species can damage biomolecules such as proteins, lipids, and DNA [4]. It is a process associated with various pathologies, including cardiovascular and neurodegenerative diseases, and is notably linked to chronic inflammation [5,6,7].

In the context of inflammation, cyclooxygenase (COX), also known as prostaglandin synthase (PGH), is a membrane-bound, bifunctional enzyme that catalyzes two key reactions: the conversion of arachidonic acid to prostaglandin G2 (PGG2) through its cyclooxygenase activity and the transformation of PGG2 to prostaglandin H2 (PGH2) via its peroxidase activity. Both processes are crucial, representing the rate-limiting step in the biosynthesis of biologically active and physiologically important prostaglandins (PGs) [8].

Two isoforms of COX have been identified: COX-1 and COX-2. COX-1 is constitutively expressed in various tissues and plays essential physiological roles, such as protecting the gastrointestinal mucosa and regulating renal blood flow. In contrast, COX-2 is induced in response to pro-inflammatory stimuli and is responsible for producing pro-inflammatory prostaglandins [8,9]. These prostaglandins amplify the inflammatory response and simultaneously generate reactive oxygen species (ROS), exacerbating oxidative stress and establishing a reciprocal relationship between chronic inflammation and oxidative damage [10,11]. Additionally, COX-2 is implicated in the development and progression of chronic inflammatory diseases such as arthritis, hypertension, neurodegeneration, and diabetes [9,12].

Traditional nonsteroidal anti-inflammatory drugs (NSAIDs) inhibit COX-1 and COX-2, effectively alleviating pain and inflammation by suppressing prostaglandin production. However, COX-1 inhibition can lead to significant side effects, such as gastric ulcers and gastrointestinal bleeding, due to interference with its protective functions. Consequently, the development of selective COX-2 inhibitors has emerged as a safer therapeutic strategy [8]. This approach targets only the enzyme responsible for inflammation, thereby avoiding the adverse effects associated with COX-1 inhibition [9].

On the other hand, antioxidant compounds play a crucial role in breaking this cycle of inflammation and oxidative stress [13,14]. These endogenous or exogenous compounds help neutralize reactive species, restoring the redox balance and reducing tissue damage associated with oxidative stress [15,16]. Among exogenous antioxidants, flavonoids in fruits, vegetables, and medicinal plants have garnered particular interest [17,18,19], not only for their wide range of activities, including notable antioxidant properties [20,21,22], but also because some flavonoids can inhibit COX-2 activity, acting as natural anti-inflammatory agents and providing dual benefits in controlling chronic inflammation [23,24].

Although the antioxidant and anti-inflammatory properties of flavonoids and other phenolic compounds have been well studied in many cases [25,26], further exploration is needed, particularly concerning the oxidation products generated by agents like AAPH (2,2’-azobis(2-amidinopropane) dihydrochloride). These compounds are widely used in methodologies to determine the antioxidant capacity of phytochemicals and synthetic compounds [27,28,29]. AAPH is a water-soluble oxidizing agent that allows precise control over oxidation rates by adjusting temperature and concentrations. It generates alkoxyl (RO^•^) and peroxyl (ROO^•^) radicals, which contribute to oxidative stress and may affect redox signaling, damaging biological macromolecules [30].

Recent studies have shown that, under certain conditions, flavonoid oxidation products can maintain or even enhance their antioxidant properties. Speisky et al. [31] investigated the antioxidant properties of quercetin and its oxidation products obtained in an alkaline medium. Using assays such as oxygen radical absorbance capacity (ORAC), Folin–Ciocalteu (FC), and ferric reducing antioxidant power (FRAP), they found that the quercetin oxidation mixture retained 93%, 77%, and 77% of its antioxidant activity, respectively, compared to non-oxidized quercetin. Furthermore, in cytoprotection studies, oxidized quercetin exhibited complete protection against the pro-oxidant and cell-lytic effects of indomethacin at a concentration ten times lower than that required for quercetin (500 nM) [32].

In line with these findings, Atala et al. [33] studied the oxidation of quercetin and 13 other structurally related flavonoids, discovering that the oxidation products lacked pro-oxidant effects and maintained antioxidant properties comparable to those of the original compounds. In particular, flavonols such as quercetin, didesoxyquercetin, isorhamnetin, and fisetin almost entirely preserved their original activity in ROS scavenging [33].

Fuentes et al. expanded this research by examining the oxidation of quercetin and the antioxidant and cytoprotective activities of the oxidation products in HS68 and Caco-2 cells. They found that these oxidative metabolites exhibited up to 20 times higher antioxidant and cytoprotective potency than quercetin [34]. They identified one oxidation product, 2,5,7,3′,4′-pentahydroxy-3,4-flavandione (BZF-Quer), demonstrating antioxidant and cytoprotective effects with a potency 200 times greater than quercetin. Additionally, this compound inhibited NF-kB (Nuclear Factor kappa-light-chain-enhancer of activated B cells) activation induced by non-steroidal anti-inflammatory drugs (NSAIDs) in Caco-2 cells at a concentration of 100 nM [27,28].

Subsequently, Speisky et al. studied the oxidation of quercetin and kaempferol and the BZFs derived from these flavonoids. They found that BZFs required significantly lower concentrations to achieve antioxidant effects comparable to their precursors. The BZF derived from kaempferol (BZF-Kae) demonstrated the ability to inhibit NF-kB activation at a concentration 100 times lower than BZF-Quer. This study also revealed that the antioxidant capacity of the quercetin and kaempferol oxidation products was exclusively attributable to the BZFs, as the products completely lost their antioxidant capacity when the BZFs were extracted [35].

Despite these studies’ valuable insights, the anti-inflammatory potential of flavonoid oxidation products remains underexplored, particularly regarding their effect on COX-2 expression. The present study aimed to evaluate the antioxidant capacity of oxidation products derived from three flavonols under controlled pH and solvent conditions using AAPH as an oxidizing agent. Furthermore, the potential anti-inflammatory properties of these compounds were investigated through COX-2 expression analysis in RAW 264.7 macrophages stimulated with lipopolysaccharide (LPS). This analysis was complemented by molecular docking and molecular dynamics simulations to explore the interaction between flavonol-derived oxidation products and the COX-2 enzyme. Additionally, density functional theory (DFT) calculations were performed to gain insights into these oxidation products’ electronic structure and stability, providing a comprehensive understanding of their antioxidant and anti-inflammatory mechanisms.

## 2. Materials and Methods

Reagent-grade chemicals were purchased from Sigma-Aldrich Chemical Co. (St. Louis, MO, USA), and solvents were purchased from Merck (Darmstadt, Germany).

The fluorescence and absorbance were measured with a Varioskan™ LUX multidetection microplate reader from ThermoFisher Scientific (Waltham, MA, USA), using white polystyrene 96-well plates by fluorescence and transparent polystyrene 96-well plates by absorbance, purchased from Nunc (Roskilde, Denmark). For the ORAC-FL and ORAC-PGR techniques, the fluorescence was read from the top, with an excitation wavelength of 485/20 nm and an emission filter of 528/20 nm, and the absorbance was read from the bottom at 525 nm, respectively. The plate reader was controlled by SkanIt™ software 7.0.2.

### 2.1. Chemical Section

#### 2.1.1. Obtaining Oxidation Products

The flavonol at 1000 mg/L was mixed with 10 molar equivalents of AAPH in a 4:1 acetonitrile: water (pH 2.0, adjusted with 1 M HCl) or methanol: water (pH 7.0, adjusted with 1 M NaOH) solution. The mixture was stirred at 60 °C for 1 h and then cooled to room temperature. The solvent was evaporated using a rotary evaporator, and the aqueous phase was washed with ethyl acetate to extract the organic phase and remove residual AAPH. The organic phase was dried, and the solid was stored at −80 °C for subsequent analysis.

#### 2.1.2. HPLC-DAD Analysis

The substrates were analyzed using an HPLC equipped with an autosampler and a diode array detector (Agilent Technologies, Santa Clara, CA, USA). Separation was achieved using a mobile phase mixture of (A) acetonitrile and (B) 2% acetic acid, with the following HPLC gradient program: 0–6 min, 2% A; 6–20 min, 15% A; 20–30 min, 25% A; 30–35 min, 40% A; 35–40 min, 50% A; 40–45 min, 75% A; 45–50 min, 85% A; 50–55 min, 95% A; 55–60 min, 100% A. The column used was an Ascentis C18, with a 15 cm × 10 mm internal diameter and a 5 µm particle size. The flow rate was set at 1 mL/min, with the column oven maintained at 25 °C.

#### 2.1.3. UPLC-ESI-MS/MS Analysis

Substrate analysis was performed on a UPLC-QTOF-ESI-MS system (Waters Xevo G2-XS QTof/Tof, Milford, MA, USA) under the following chromatographic conditions: mobile phase A: 0.1% formic acid; mobile phase B: acetonitrile or methanol, with the following UPLC gradient program: 0–3 min, 100% A; 3–30 min, 10% A. The column used was an ACQUITY UPLC HSS T3 1.8 µm; 2.1 × 100 mm, with a flow rate of 0.2 mL/min. The mass spectrometer operated in negative ion mode, and data were acquired in continuous mode at 0.5 s intervals. The low-energy collision energy was set to 10 V, while high-energy collisions were ramped from 10 to 75 V. The cone and capillary voltages were 40 V and 0.53 kV, respectively, and the desolvation gas flow rate was 993 L/h at 500 °C.

### 2.2. Biological Section

#### 2.2.1. ORAC-FL

Fluorescein (Fl) solutions (FL, 70 nM, final concentration) were combined with the respective substrates dissolved in methanol or acetonitrile (0.01–1 mg/L) in each well of a plate. The mixture was pre-incubated at 37 °C for 15 min, followed by the addition of a solution of AAPH (18 mM, final concentration). The plate was then placed in the reader, which automatically agitated the mixture before reading. Fluorescence was recorded every minute until the fluorescein signal was depleted. A blank containing FL and AAPH with methanol or acetonitrile instead of the test solution was included in each assay [36].

Additionally, five calibration solutions of commercial Trolox (1.25–5.00 mg/L) were used as positive controls. The antioxidant capacity was expressed as ORAC-FL index values and was quantified by integrating the area under the fluorescence decay curve (AUC) for each substrate relative to Trolox (Equation (1)). All reaction products were prepared in triplicate, and at least three independent assays were conducted for each sample.

ORAC Index = (AUC substrate)/(AUC Trolox)(1)

#### 2.2.2. ORAC-PGR

Pyrogallol red (PGR) solutions (PGR 70 µM final concentration) were combined with substrates dissolved in methanol or acetonitrile (1–120 mg/L) in each well of a plate. The mixture was pre-incubated at 37 °C for 15 min and added to AAPH solution (800 mM final concentration). The plate was immediately placed in the reader, and the mixture was automatically agitated before reading. Absorbance was recorded every minute until it dropped to <0.1. A blank containing PGR and AAPH with acetonitrile or methanol instead of the test solution was used in each assay. Five calibration solutions of Trolox (3.75–50 mg/L) were also included as positive controls. Antioxidant capacity was expressed similarly to the ORAC-FL index (Equation (1)). All reaction products were prepared in triplicate, and at least three independent assays were performed for each sample [36,37].

#### 2.2.3. Viability Assay

Human foreskin fibroblast (HFF) (ATCC SCRC-1041) cells were cultured in DMEM supplemented with 10% inactivated fetal bovine serum (FBSi) and 1% antibiotics (penicillin–streptomycin). In this culture and all others described below, the cells were incubated at 37 °C, with 5% CO_2_ and 98% relative humidity.

Murine macrophage (RAW 264.7) cells (ATCC^®^ TIB-71™) are murine macrophages (BALB/c strain). Cells were cultured on RPMI 1640 with L-glutamine medium (Biological Industries, Kibbutz Beit HaEmek, Israel) supplemented with 5% *v*/*v* FBSi, 100 U/mL penicillin, and 100 mg/mL streptomycin at 37 °C, 5% CO_2_, and 98% relative humidity.

The cell viability assay was performed using the tetrazolium salt reduction method (MTT) with 3-(4,5-dimethylthiazol-2-yl)-2,5-diphenyltetrazolium bromide. Cells were cultured in 96-well plates at a density of 5 × 10^4^ cells/well and incubated at 37 °C with 5% CO_2_ for 24 h in the appropriate culture medium. After cell adhesion, the medium was replaced with 100 µL of fresh medium containing the compounds of interest (flavonols or oxidation products) at concentrations of 10 and 100 mg/L, and the cells were incubated for an additional 24 h. Subsequently, the cells were washed three times with PBS to remove residual compounds. The medium was then replaced with phenol red-free culture medium, and 10 µL of MTT dye (5 mg/mL) was added to each well. Cells were incubated for 4 h at 37 °C to allow the formation of formazan crystals, which were dissolved by adding DMSO. The plates were kept for 1 h at 37 °C, and the optical density (OD) was measured at 570 nm using a Thermo Scientific™ Varioskan Flash microplate reader. Under these conditions, OD is directly proportional to the number of viable cells in each well [38].

#### 2.2.4. Cellular Antioxidant Capacity (CAA) of Flavanols and Oxidation Products

HFF cells were cultured in DMEM supplemented with 5% fetal bovine serum in 96-well plates at a density of 5 × 10^4^ cells/well and incubated at 37 °C with 5% CO_2_ for 24 h. After attachment, the medium was replaced with 100 µL of culture medium containing 25 µM DCFH-DA and incubated for 1 h under the same conditions. Subsequently, the cells were washed three times with PBS to remove the excess probe. The cells were then exposed to 100 µL of a solution containing 600 µM AAPH and antioxidant compounds (parent flavonols or oxidation mixtures) diluted in a culture medium at 10 µg/mL. Controls included cells treated with AAPH only (control AAPH), cells treated with antioxidants without AAPH (autofluorescence control), and untreated cells (blank). Fluorescence was measured using a Thermo Scientific™ Varioskan Flash microplate reader with an excitation wavelength of 485 nm and an emission wavelength of 538 nm at 37 °C every 5 min for 1 h. The AUC of fluorescence versus time was calculated for each condition after blank subtraction, thus quantifying the antioxidant activity [35,39]:

CAA = 100 − (AUC substrate/AUC control AAPH) × 100%(2)

#### 2.2.5. Determination of COX-2 Expression by Western Blot

Murine macrophages RAW 264.7 were cultured as previously described. For the experiments, cells were seeded at a density of 5 × 10^5^ cells/well in six-well plates and incubated overnight. They were then exposed to antioxidants and oxidation products at 100 and 10 mg/L concentrations, respectively, for 48 h. Afterward, cells were washed with PBS and incubated in fresh medium for 24 h. Finally, lipopolysaccharide (LPS) (1 μg/mL) was added to induce inflammation, followed by an additional 24 h incubation [40].

Cells were washed with PBS and lysed using RIPA buffer for protein isolation, followed by sonication and incubation on ice. Lysates were centrifuged, and total protein concentration was determined using the micro-BCA kit (Thermo Scientific, Waltham, MA, USA). Proteins (50 μg) were separated by electrophoresis on 10% polyacrylamide gels and transferred onto nitrocellulose membranes, blocked with 3% BSA in PBST. Membranes were incubated with a primary antibody against COX-2 (ab52237, Abcam, Cambridge, UK) and, after washing, with an HRP-conjugated anti-rabbit IgG secondary antibody (ab6721, Abcam). Detection was performed using chemiluminescence with the Immobilon^®^ Classico substrate (Sigma-Aldrich, St. Louis, MO, USA) [41].

For loading control, membranes were treated with a stripping solution, re-blocked, and incubated with a primary antibody against β-actin (ab3280, Abcam) and an HRP-conjugated anti-mouse IgG secondary antibody (ab6728, Abcam). Bands were analyzed using densitometry with ImageJ software v1.53.

#### 2.2.6. Statistical Analysis

Statistical analysis was performed using GraphPad Prism 9.5.0 (GraphPad Software, San Diego, CA, USA). Data are presented as the mean of three independent experiments. One-way ANOVA followed by Dunnett’s post-test was used for statistical analysis, with *p* < 0.05 considered statistically significant.

### 2.3. Computational Studies

#### 2.3.1. DFT Calculations

All structures were optimized in their neutral, cationic, and anionic state at the r2SCAN-D4/def2-TZVPP composite and M06-2X density-functional level of theory [42]. COSMO (e = 80) [43] was employed to model solvent effects. At the same time, the RI approximation was used to speed up calculations [44]. Energies were obtained at the same level. From these energies, ionic potentials and electron affinities were obtained. All these calculations were performed with TURBOMOLE v7.7 [45].

For electronic-structure analysis of the radicals, single points were performed on the cationic systems, employing the same method, with Orca 5.0 [46] CPCM, with the COSMO epsilon function used for solvation effects. The resulting orbitals were analyzed with NBO 7.0.

The ionization energy (*IE*) and electron affinity (*EA*) were calculated adiabatically [47], where *IE* = Ecation − Eneutral and *EA* = Eneutral − Eanion. The calculation of the bond dissociation energy of Wiberg (BDEŋW) followed the method outlined by Rakiba Rohman et al., resulting in BDE as a product of η and the Wiberg bond index [48]. It is important to note that the BDEsnW value is a qualitative approximation to identify the most labile hydrogen and should not be interpreted as an exact thermodynamic value.

Chemical hardness was calculated according to the definition proposed by Parr and Pearson [49]. To achieve symmetry to ensure electronegativity symmetry, the product was multiplied by half, as highlighted by Pearson [50]:

(3) $$\eta ={\left(\frac{\partial^{2}E}{\partial^{2}N}\right)}_{v\left(r\right)}\approx \frac{IE-EA}{2}$$

In addition, as a local reactivity descriptor, we used the Fukui radical function $f^{0}\vec{\left(r\right)}$. The radical Fukui function used in our analysis was obtained following the procedure described by Osorio et al., where the consensus value of the Fukui function $f_{k}^{0}$ is obtained for each atom [51]. The chemical interpretation $f_{k}^{0}$ is that higher values of this descriptor represent regions in a molecule more susceptible to free radical attack.

#### 2.3.2. Molecular Docking

To predict the structure of each COX-2–ligand complex, comprehensive molecular docking simulations were performed using a COX-2 monomer and the compounds A1, A2, A3, BZF-Quer-OH, BZF-Kaem-OH, and BZF-Ram-OH, including celecoxib as the control. The crystallographic structure of COX-2 (PDB ID: 3LN1) [52] was obtained from the Protein Data Bank [53]. Autodock-GPU software 1.5.3 was employed for the docking calculations, with the grid box centered on the active site of celecoxib, using dimensions of 30 × 30 × 30 Å and a grid spacing of 0.375 Å. Each docking simulation generated 10 conformations, and the highest-scoring structures were selected for further molecular dynamics simulations.

#### 2.3.3. Molecular Dynamics Simulations and MM-GBSA Calculations

To further explore the interaction between COX-2 and the ligands, all-atom classical molecular dynamics (MD) simulations were performed. The COX-2 dimer was prepared, with the ligand in the second monomer placed in the same conformation as that of monomer A. Protonation states for the system were set at pH 7.0 using the H++ web interface [54]. Ligand−protein complexes were constructed using the previously docked structures. Ligand parameters were derived from GAFF2, with charges calculated via the AM1-BCC semi-empirical method using the antechamber package from AmberTools24. The ff14SB forcefield was used to model the protein structures. MD simulations were executed with an 8.0 Å cutoff for nonbonded interactions, utilizing the particle mesh Ewald method to describe long-range electrostatics and the SHAKE algorithm to constrain hydrogen-involved bonds. These MD simulations were run using the pmemd.CUDA program from Amber24 with GPU acceleration. The simulation protocol included (a) 5000 steps of steepest descent minimization, followed by 3000 conjugate gradient minimization steps for the entire system, (b) 1.0 ns of NVT heating from 0 to 300 K, and (c) 100 ns of unrestrained NPT production dynamics at 300 K and 1 bar.

Binding free energies between the protein and ligand were calculated using the Molecular Mechanics Generalized-Born Surface Area (MM-GBSA) method, including 1250 frames extracted from the final 50 ns of each MD simulation. These calculations employed the parallel MMPBSA.py.MPI module from AmberTools24, following a single trajectory method. The modified GB model (igb = 5) with mbondi2 parameters accounted for the polar solvation-free energy, applying α, β, and γ values of 1.0, 0.8, and 4.85, respectively. The dielectric constants were set to 80 for the solvent and 1 for the protein. An ionic strength of 0.15 M was also incorporated into the energy calculations to reflect physiological conditions.

## 3. Results

### 3.1. Chemical Section

#### 3.1.1. Obtaining Oxidation Products

The oxidation of three flavonols—quercetin (A1), kaempferol (A2), and rhamnetin (A3)—was carried out using AAPH as an oxidizing agent. To optimize the reaction conditions and facilitate the generation of oxidation products, quercetin was used as a model compound. Several parameters were systematically evaluated, including pH (2 and 7), solvent nature (protic: MeOH; aprotic: ACN), temperature (37 and 60 °C), reaction time (0, 30, 60, and 120 min), and AAPH concentration (10 and 20 equivalents).

The primary objective was to identify potential oxidation products corresponding to benzofuranones (BZFs) derived from flavonols. These BZFs are characterized by an absorption maximum at 290 nm and exhibit greater polarity compared to their flavonol precursors. Therefore, particular attention was given to chromatographic peaks displaying this absorption maximum and shorter retention times than their parent compounds, ensuring precise identification of the oxidation products of interest [30,47].

**Figure 1:**
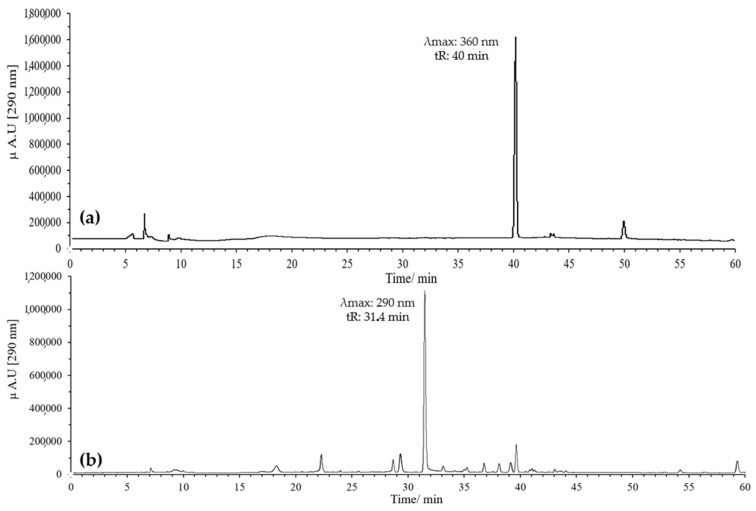
Optimization of the oxidation temperature of quercetin at pH 7 in MeOH, 120 min, 20 eq of AAPH: (**a**) 37 °C; (**b**) 60 °C.

The oxidation reaction was optimized by evaluating two temperatures, 37 °C and 60 °C, with monitoring for up to 120 min in both cases. At 37 °C, quercetin oxidation was very slow, and even after 120 min, a significant amount of unreacted compound remained, indicating that this temperature was not suitable for efficient flavonol oxidation (Figure 1). In contrast, at 60 °C, oxidation was more efficient, leading to the complete disappearance of quercetin in the same period and the formation of a peak with the expected characteristics for BZF (tR = 31.4 min, λ_max = 290 nm).

**Figure 2:**
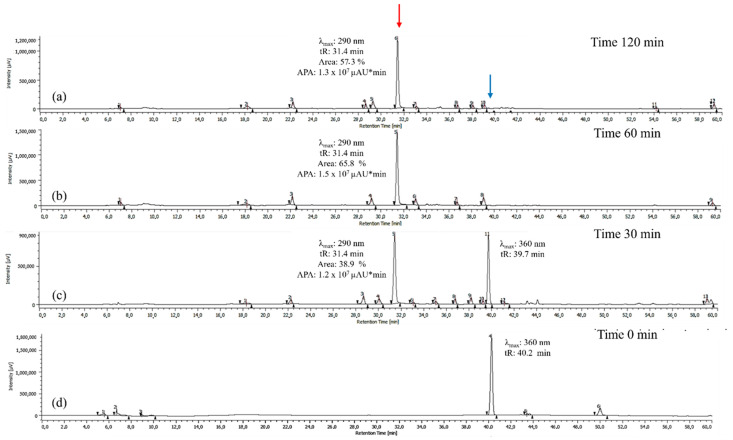
Chromatograms of the quercetin oxidation products at 60 °C, 10 eq of AAPH, pH 7 in MeOH: (**a**) 120 min; (**b**) 60 min; (**c**) 30 min; (**d**) 0 min. The BZF peak is highlighted in red, and the quercetin peak is highlighted in blue. The areas shown correspond to the BZF peak, which exhibits a larger area at 60 min and a maximum absorption at 290 nm. APA corresponds to the absolute peak area for the BZF peak in µAU*min.

Once 60 °C was established as the optimal temperature, the reaction time was optimized by maintaining this temperature constant and evaluating reaction times of 30, 60, and 120 min. As shown in Figure 2, the peak potentially corresponding to BZF (tR = 31.4 min, λ_max = 290 nm) increases significantly over time in both absolute peak area (APA) and percentage area, indicating its preferential formation under these conditions. At 30 min, the BZF peak emerges with an APA of 1.2 × 10^7^ µAU*min and a percentage area of 38.9%, marking the onset of quercetin oxidation. At 60 min, the APA reaches its maximum (1.5 × 10^7^ µAU*min), while the percentage area increases to 65.8%, confirming that this is the optimal reaction time for BZF formation. At this point, BZF is not only produced in the highest absolute amount but also dominates relative to other metabolites.

At 120 min, the APA slightly decreases (1.3 × 10^7^ µAU*min), and the percentage area drops to 57.3%, suggesting the occurrence of possible side reactions that may affect BZF stability. These results confirm that 60 min is the optimal reaction time, maximizing BZF production both in absolute terms and in proportion to other oxidation products.

**Figure 3:**
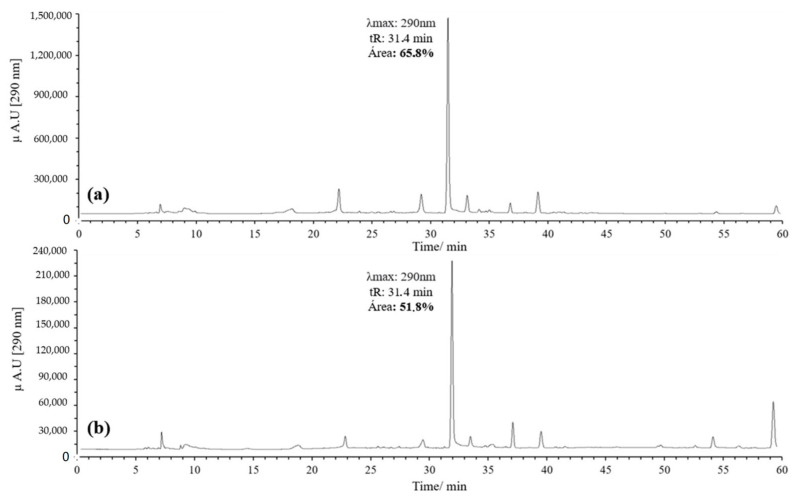
Chromatograms of the quercetin oxidation products at 60 °C, 1 h, pH 7 in MeOH: (**a**) 10 eq of AAPH; (**b**) 20 eq of AAPH.

Initially, 20 equivalents (eq) of AAPH were used, following the procedure reported by Venkat et al., in which AIBN was employed as the oxidizing agent [30]. However, the concentration of AAPH was subsequently reduced to 10 eq to reduce reagent consumption and simplify handling. A higher percentage of area was observed with 10 eq, leading to the decision to use this concentration (Figure 3).

**Figure 4:**
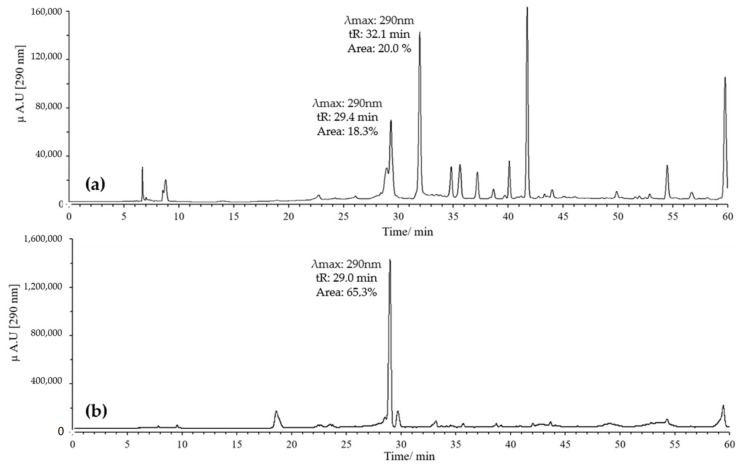
Chromatograms of quercetin oxidation mixture (MOx) at 60 °C, 10 eq of AAPH, 60 min, and pH 2. (**a**) MeOH; (**b**) ACN.

On the other hand, combinations of pH 2 with ACN and pH 7 with MeOH were evaluated. Generally, it was found that when combining pH 2 with MeOH, quercetin exhibited several peaks, with two prominent ones potentially corresponding to the derivatives of interest. These had retention times of 29 and 32 min, with maximum absorption at 290 nm; the peak at 32 min showed the highest intensity (Figure 4a).

**Figure 5:**
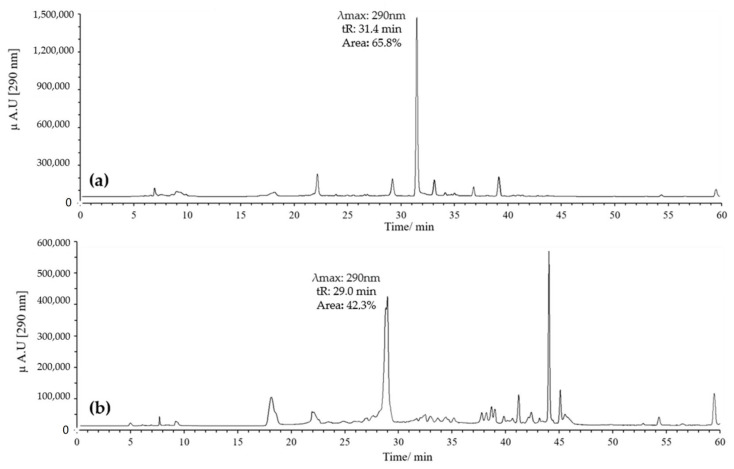
Chromatograms of quercetin MOx at 60 °C, 10 eq AAPH, pH 7 for 60 min. (**a**) MeOH; (**b**) ACN.

In contrast, combining pH 2 with ACN resulted in a high-intensity peak at 29 min with absorption at 290 nm, without the formation of the peak at 32 min (Figure 4b). Conversely, using pH 7 with MeOH yielded a single high-intensity peak at 32 min with absorption at 290 nm, while the peak at 29 min disappeared (Figure 5a). Finally, employing pH 7 with ACN showed similar behavior to that observed for pH 2 with ACN, but the intensity and resolution of the peak at 29 min were affected (Figure 5b).

**Figure 6:**
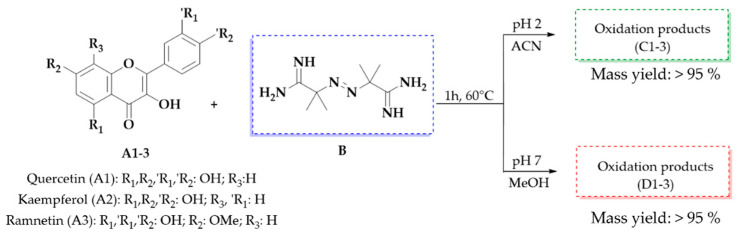
The oxidation reaction of flavonols A1–3 with 10 equivalents of AAPH (B) at 60 °C for 1 h, pH 2 (products C1–3), and pH 7 (products D1–3) and the mass yield of the oxidation mixture obtained.

Based on these results, we decided to work with two specific combinations of pH and solvent: pH 2 with ACN, which favors the appearance of the peak at 29 min, and pH 7 with MeOH, which promotes the appearance of the peak at 32 min, both with absorption at 290 nm. Notably, the mass yield of the oxidation mixtures consistently remained above 95% under all conditions evaluated in this study, demonstrating the efficiency of the reaction under the selected parameters (Figure 6).

#### 3.1.2. HPLC-DAD and UPLC-Ms/Ms Analysis of Oxidation Products

The oxidation products obtained were analyzed using HPLC-DAD and HPLC-MS/MS. In all cases, complete disappearance of the precursors was ensured before proceeding with subsequent analyses. Once the products were formed, they were stored at −80 °C. Before use, their safety was confirmed through HPLC-DAD.

**Figure 7:**
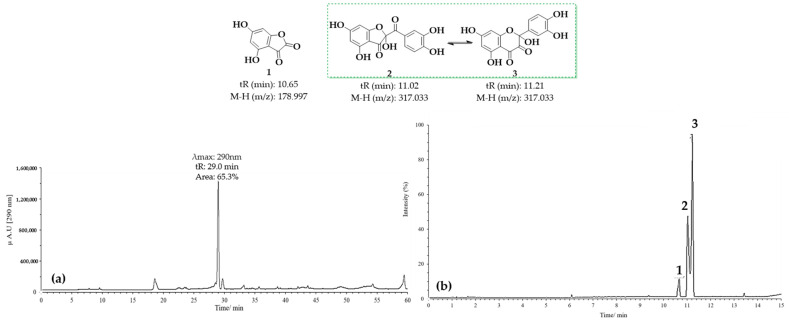
Chromatograms of MOx quercetin pH 2. (**a**) HPLC-DAD; (**b**) UPLC-Q-TOF.

Figure 7 presents the results obtained for the oxidation product C1, derived from quercetin at pH 2 in the presence of acetonitrile. Figure 7a shows the HPLC-DAD chromatogram recorded at a wavelength of 290 nm, where a prominent peak with a retention time (tR) of 29 min is observed. This peak may correspond to 2-(3,4-dihydroxybenzoyl)-2,4,6-trihydroxybenzofuran-3(2H)-one (BZF-Quer-OH), which is known to have an absorption maximum at 290 nm. On the other hand, Figure 7b displays the chromatogram obtained from the UPLC-Q-TOF analysis, where three peaks with mass-to-charge ratios (*m*/*z*) of 178.9972 (tR: 10.65 min), 317.0328 (tR: 11.02 min), and 317.0328 (tR: 11.21 min) can be observed, and these are tentatively assigned to the structures depicted in Figure 7 [28,29].

**Scheme 1:**
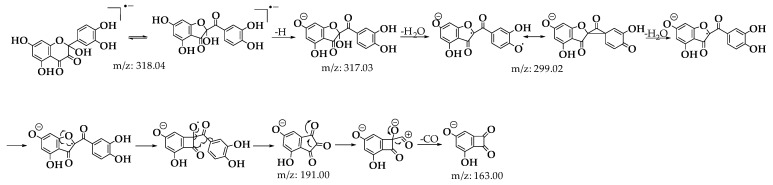
Fragmentation patterns for signals 2 and 3 of the C1 oxidation product.

Upon analyzing the mass spectra of the signals labeled 2 and 3 (Figures S1 and S2), peaks were observed at *m*/*z*: 318.0323 (M-), 317.0328 (M-H), and 299.0187 (M-H_2_O), consistent with the findings described by Fuentes et al. [28] for the BZF-Quer-OH structure (Scheme 1) [28,30,35]. Notably, the formation of a molecular ion was detected, which is uncommon in tandem mass spectrometry analyses using negative ESI coupled with collision-induced dissociation (CID). Typically, the most common fragments are of the type [M−nH]ⁿ^−^. However, previous studies have shown that for molecules with high electron affinity, forming a negative radical anion is possible during this type of analysis [55,56,57,58,59,60]. Additionally, it was noted that signals 2 and 3 exhibit similar fragmentation patterns, suggesting that one of these could be the BZF-Quer-OH derivative, while the other corresponds to its isomer, 2-(3,4-dihydroxyphenyl)-2,5,7-trihydroxychromane-3,4-dione. Furthermore, it is proposed that signal 1 observed in the chromatogram of Figure 7 likely corresponds to a derivative of BZF-Quer-OH, where the C ring acted as a reactive center for the formation of 4,6-dihydroxybenzofuran-2,3-dione (Figure S3).

**Figure 8:**
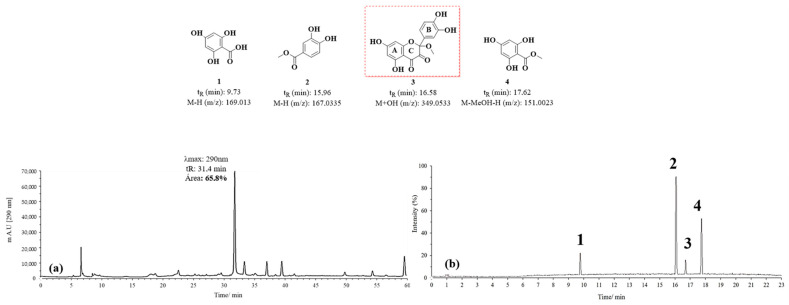
Chromatograms of MOx quercetin pH 7 (**D1**). (**a**) HPLC-DAD; (**b**) UPLC-Q-TOF.

The chromatograms obtained by HPLC-DAD and UPLC-Q-TOF for the D1 oxidation product are shown in Figure 8. Figure 8a highlights an intense signal with a maximum absorption at 290 nm. However, unlike the signal observed in Figure 7, this one shows a retention time (tR) of 32 min, suggesting the formation of a compound with lower polarity than the previously mentioned BZF-Quer-OH derivative. In Figure 8b, signals corresponding to *m*/*z* values of 169.013 (tR: 9.73 min), 167.0335 (tR: 15.96 min), 349.0533 (tR: 16.58 min), and 151.0023 (tR: 17.62 min) are identified. The proposed structures for these signals were obtained based on the mass spectra analyzed (Figures S4–S7).

**Scheme 2:**
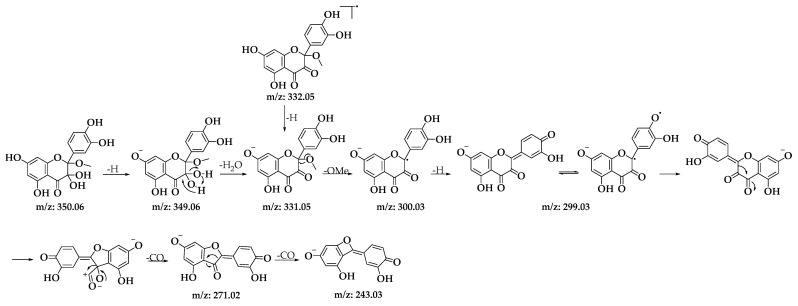
Fragmentation patterns for signal 3 of the D1 oxidation product.

Among the structures analyzed, signal 3 stands out due to its *m*/*z* ratio, which suggests the presence of a methoxylated analog of BZF-quercetin (BZF-Quer), specifically 2-(3,4-dihydroxyphenyl)-5,7-dihydroxy-2-methoxychroman-3,4-dione (BZF-Quer-OMe). This finding is supported by a fragmentation pattern with the following *m*/*z* values: 350.0599 (M + H₂O), 349.0533 (M + H₂O − H), 332.0470 (M-), 331.0443 (M − H), and 300.0242 (M − MeOH). These values match those reported by Venkat et al., who performed quercetin oxidation using AIBN as a radical initiator and observed the formation of this methoxylated analog (Scheme 2) [30].

**Scheme 3:**
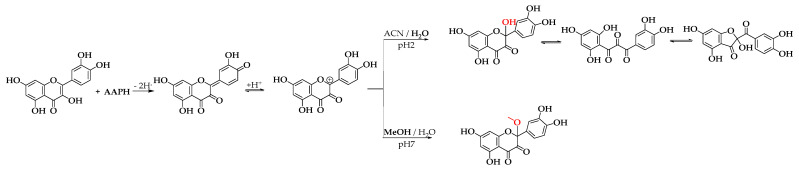
Proposed mechanism for the formation of BZF-Quer-OH under acidic conditions (pH 2, ACN/H₂O) and BZF-Quer-OMe under neutral conditions (pH 7, MeOH/H₂O). The red-highlighted atoms indicate the structural differences between the two species formed, corresponding to the hydroxyl group in BZF-Quer-OH and the methoxy substituent in BZF-Quer-OMe.

Scheme 3 illustrates the proposed mechanism for forming BZF-Quer-OH and its analog BZF-Quer-OMe. The mechanism begins with the abstraction of a hydrogen atom from position 4’ of the B ring of quercetin, forming an ortho-hydroquinone. After protonation, a carbocation is generated at position 2 of the C ring of quercetin. Depending on the oxidation conditions in an acidic medium (pH 2) and with a polar aprotic solvent, adding water is favored, forming BZF-Quer-OH. However, in a neutral medium (pH 7) and with a polar protic solvent, adding MeOH is preferred over water, which aligns with the basicity of the species involved.

**Figure 9:**
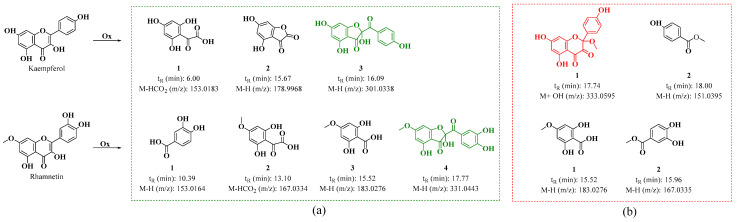
Proposed oxidation metabolites for the oxidation products of kaempferol and rhamnetin, based on their fragmentation patterns at (**a**) pH 2 with ACN and 10 eq of AAPH and (**b**) pH 7 with MeOH and 10 eq of AAPH mechanism for the formation of BZF-Quer-OH in the presence of pH 2/ACN and BZF-Quer-OMe in the presence of pH 7/MeOH.

On the other hand, in the oxidation products **C2** and **D2** obtained from the oxidation of kaempferol in acidic and neutral media, metabolites similar to those found for quercetin were observed. Figure S8a shows the HPLC-DAD chromatogram, which displays a strong signal with maximum absorption at 290 nm. This signal may correspond to 2,4,6-trihydroxy-2-(4-hydroxybenzoyl)benzofuran-3(2H)-one (BZF-Kaem-OH) [35]. This is evidenced by the UPLC-Q-TOF chromatogram (Figure S8b), where three metabolites are identified (Figure 9 and Figures S9–S11). Among them, signal 3 shows a fragmentation pattern that matches the proposed structure for BZF-Kaem-OH, with *m*/*z* 302.0397 (M-) corresponding to the molecular ion, *m*/*z* 301.0337 (M-H) corresponding to the base peak due to the loss of a hydrogen atom at position 4’ of ring B, and *m*/*z* 274.0452 (M-OH) corresponding to the loss of the hydroxyl group at position 2 of ring C (Figure S11).

In the **D2** and **D1** products, the HPLC-DAD shows a strong signal with maximum absorption at 290 nm, exhibiting lower polarity than BZF-Kaem-OH (Figure S12a). In the UPLC-Q-TOF (Figure S12b), the formation of a methoxylated analog of BZF-Kaem-OH is observed, identified as 5,7-dihydroxy-2-(4-hydroxyphenyl)-2-methoxychroman-3,4-dione (BZF-Kaem-OMe). This analog shows a fragmentation pattern with *m*/*z*: 334.0652 (M + H₂O), 333.0595 (M + H₂O − H), 316.0522 (M-), 315.0486 (M-H), and 284.0334 (M-MeOH) (Figure S13). In addition, another phenolic analog was identified (Figure 9 and Figure S14).

Regarding the oxidation mixtures of rhamnetin (**C3** and **D3**), the acidic mixture showed very similar behavior to that of mixtures **C1** and **C2**, with the formation of a product whose fragmentation pattern corresponds to 2-(3,4-dihydroxybenzoyl)-2,4-dihydroxy-6-methoxybenzofuran-3(2H)-one (BZF-Ram-OH) (Figures S15 and S19). Three other products were also identified, with their structures proposed based on their fragmentation patterns (Figures S16–S19). These structures are like those described by Atala et al. for quercetin, derived from the formation of species resulting from the degradation of the C ring of the parent flavonol [28,33].

Unlike the **C3** mixture, the **D3** (Figure S20) mixture does not follow the same trend observed in its analogs. In this oxidation mixture, no methoxylated analog of BZF-Ram was detected; instead, only two oxidation products are observed, as presented in Figure 9. Their structures are assigned based on their fragmentation patterns and appear to derive from the degradation of the C-ring of rhamnetin (Figures S21 and S22).

The findings above highlight that oxidation reactions involving AAPH as an oxidizing agent, generating RO^•^ and/or ROO^•^ radicals, can lead to diverse transformations of the same substance depending on its environment. In general, it was observed that for the three flavonols analyzed by UPLC-MS/MS, the combination of acidic pH and an apolar protic solvent favors the formation of BZFs-OH derivatives and other phenolic compounds. In contrast, oxidations at pH 7 and in a polar protic solvent can lead to the formation of substituted BZFs or entirely different metabolites. It is important to emphasize that understanding the degradation mechanisms of flavonoids could enable the development of more stable and effective formulations for antioxidant applications. Moreover, studying new oxidation products opens the door to exploring compounds that may exhibit novel antioxidant properties or, potentially, toxic effects, which is crucial for assessing their safety profiles in therapeutic applications.

### 3.2. Biological Section

#### Analysis of the Antioxidant Capacity and Cell Viability of Oxidation Products

Figures S24–S35 display the degradation kinetics of fluorescein (FL) and pyrogallol red (PGR) upon exposure to radicals generated by AAPH in the presence of quercetin, kaempferol, rhamnetin, luteolin, rosmarinic acid, ferulic acid and oxidation products C1–3 and D1–3. The area under the decay curve (AUC) was calculated by integrating the fluorescence decrease for FL (ORAC-FL) and the absorbance decrease for PGR (ORAC-PGR). The Trolox calibration curve is shown in Figure S23 as a reference. Generally, a degradation profile dependent on the concentrations of the analyzed samples was observed. Despite being free of the initial precursor, all the oxidation products demonstrated antioxidant capacity in the ORAC-FL assay. However, only the C1–3 products and D2 exhibited antioxidant capacity in the ORAC-PGR assay.

It is important to mention that using FL and PGR as probe molecules in ORAC assays is based on their ability to provide different information regarding antioxidant capacity. The ORAC-FL index is primarily related to the stoichiometric efficiency of antioxidants, which measures the number of radicals neutralized compared to a standard such as Trolox. This is evidenced by the decay curves and AUC values, where longer induction times are associated with low reactivity of the probe in its reaction with radicals. In contrast, the ORAC-PGR index provides information about the reactivity of antioxidants with free radicals, where short or absent induction times indicate high reactivity of the probe with the radicals present in the medium [36,61].

Furthermore, it has been found that the ORAC index value depends on the reactivity of RO• and ROO• radicals and that the contribution of these species varies depending on the reactivity of the probe and the antioxidant. This variability interprets ORAC results as complex, especially in the case of compound mixtures [62,63]. This observation is consistent with recent studies, such as that of Pozo-Martinez et al., who suggest that the ORAC-PGR assay may be insufficient to determine the antioxidant capacity of complex mixtures due to the variability in the reactivity of the metabolites present in such products. Therefore, this could indicate that, although the metabolites obtained through the different oxidation products have antioxidant capacity in stoichiometric terms, it is possible, at least for those that did not yield results in the ORAC-PGR assays, that the combination of different metabolites in D1 and D3 results in a net reactivity of the mixture that is insufficient to prevent the oxidation of the PGR probe [36].

Likewise, in this study, the cellular antioxidant activity (CAA) and cytotoxicity of flavonol oxidation products, as well as their precursors, were evaluated at a concentration of 10 µg/mL in HFF cells (Table 1 and Figures S36–S38). In this context, the oxidation products obtained at pH 2, such as Mox-Kam pH 2 and Mox-Quer pH 2, exhibited the highest CAA values, reaching 93.3 ± 2.3% and 91.2 ± 0.4%, respectively, compared to the oxidation products obtained at pH 7. These results align with the trend observed in ORAC assays. Furthermore, it was noted that the CAA of most oxidation mixtures exceeded that of the original flavonols, consistent with the findings reported by Speisky et al. [35]. This result is particularly relevant, as it highlights that the type of assay used significantly influences the evaluation of antioxidant capacity. While ORAC assays predominantly assess the chemical reactivity and structural characteristics of antioxidant compounds, biological systems incorporate additional variables such as bioavailability, intracellular transport, subcellular localization, and interaction with enzymatic systems. This suggests that oxidation products exhibit high antioxidant capacity in chemical systems like ORAC and demonstrate superior efficacy in cellular contexts. This effect may be partially attributed to their ability to modulate intracellular antioxidant pathways.

On the other hand, it was observed that the oxidation products obtained at pH 2 and pH 7 showed higher ORAC-FL indices compared to Trolox and known antioxidants such as luteolin, rosmarinic acid, and ferulic acid. This finding suggests that the oxidation products have significant potential for developing new antioxidant compounds. However, the ORAC-PGR indices of most oxidation products were consistently lower than that of Trolox. This result does not necessarily indicate a lower antioxidant capacity. Instead, it reflects the limitations of the ORAC-PGR method, as its interpretation depends on the reactivity of the probe and the specific radicals evaluated. In the case of metabolite mixtures, interactions between compounds and alternative antioxidant mechanisms may influence the overall response, making ORAC-PGR results more complex to interpret.

Therefore, future studies should be cautious when using ORAC-PGR to evaluate complex mixtures, as the method may underestimate antioxidant capacity in systems where multiple metabolites interact. It should be complemented with other techniques to provide a more comprehensive assessment of antioxidant activity.

It has been reported that oxidation products of kaempferol and quercetin obtained via alkaline oxidation, primarily metabolites of the benzofuranone (BZF) type and/or onion extracts rich in these molecules, can activate the transcription factor Nrf2 and inactivate the NF-κB factor [35,64]. Nrf2 regulates the expression of key antioxidant enzymes, such as superoxide dismutase (SOD) and glutathione peroxidase (GPx), thereby enhancing antioxidant defenses. Simultaneously, the inactivation of NF-κB reduces the expression of prooxidant and proinflammatory genes. Thus, it is likely that the presence of BZFs in the oxidation mixtures obtained in the present study is responsible for the enhanced antioxidant capacity observed in cellular systems.

The observed shift in the trend between ORAC and CAA assays underscores that while chemical methods like ORAC are valuable tools for assessing a compound’s antioxidant capacity and structural relationships, systems that more accurately represent cellular physiology must also be considered. These systems allow for more precise analysis of a compound’s true antioxidant potential in a biological context, integrating key factors such as bioavailability and intracellular interactions.

Atala et al. suggested that, in the analysis of flavonoid antioxidant capacity, compounds generated during the assays could significantly contribute to the results, potentially leading to an overestimation of the antioxidant capacity attributed exclusively to the flavonoid under study [33]. In this work, we confirm and expand on Atala et al.’s findings, demonstrating that oxidation products generated using AAPH as a radical generator significantly impact the observed antioxidant properties. This reveals that the initial compound and its oxidation products are critical in the quantitative results obtained in ORAC assays. Therefore, we propose a more precise approach to describing antioxidant capacity in these systems, the gross antioxidant capacity (GAC), which encompasses both the original compound and its oxidation products. However, the exact contribution of the metabolites formed varies depending on the nature of the substrate and the specific oxidation conditions, such as the oxidant used, pH, and solvent. This emphasizes the inherent complexity of antioxidant compound chemistry, where multiple factors interact to determine their capacity.

Additionally, cellular viability analyses indicated that all oxidation products maintained high levels of viability, with values ≥85%. This result is particularly significant, as it demonstrates that the oxidation mixtures generated under the evaluated conditions do not exhibit relevant cytotoxic effects on HFF and RAW 264.7 cells. The preservation of cellular viability is a key indicator of the biological compatibility of the oxidation products. It suggests that the evaluated concentrations (10 µg/mL) are appropriate to maximize antioxidant activity without compromising cellular integrity. These findings are consistent with the reports of Kelly L. Wolfe, who emphasized that cellular viability assessment is a critical parameter for determining the safety of antioxidant compounds in biological systems [39]. Maintaining high cellular viability while exhibiting significant antioxidant effects reinforces that flavonol oxidation products, such as quercetin and kaempferol, possess a favorable profile for therapeutic applications, combining antioxidant efficacy with low toxic potential.

**Figure 10:**
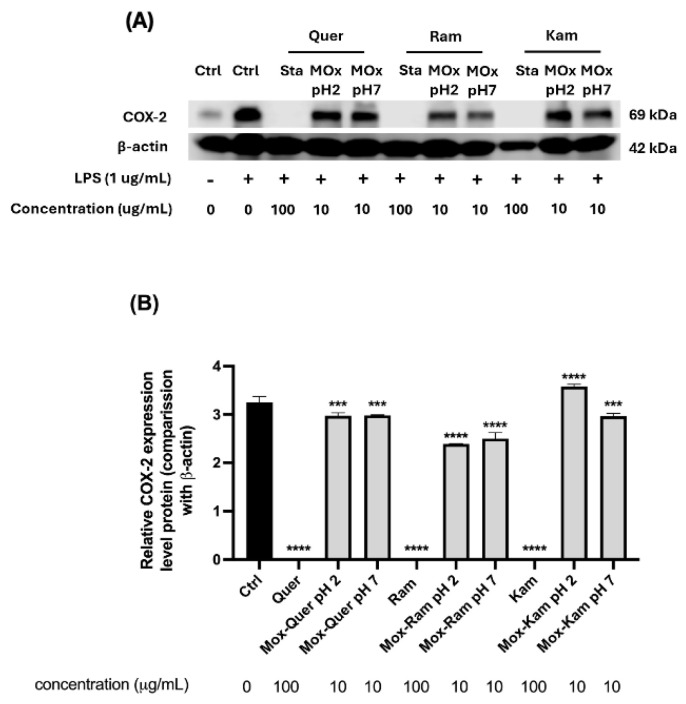
(**A**) Representative Western blot images showing COX-2 expression in RAW 264.7 macrophages treated with flavonol standards (quercetin, rhamnetin, kaempferol) and their oxidation products obtained at pH 2 and 7. Cells were stimulated with LPS (1 µg/mL) for 24 h, and treatments were applied at 10 µg/mL concentrations for oxidation products and 100 µg/mL for standards (Sta); β-actin was used as the loading control (Ctrl). (**B**) Quantification of COX-2 expression levels normalized to β-actin. Results are expressed as relative protein expression compared to LPS-treated control. One-way ANOVA with Dunnett’s post-test, *** *p* < 0.005, **** *p* < 0.0005 indicate statistically significant differences compared to the LPS-treated control.

Additionally, the inhibition of COX-2 expression was determined in RAW 264.7 macrophages stimulated with LPS, as previously described in the literature [65,66]. Earlier studies reported that low concentrations of antioxidants like quercetin could induce COX-2 overexpression, while enzyme inhibition was observed at higher concentrations (100 µg/mL) [40]. In this study, COX-2 expression was evaluated by Western blot for all samples (Figure 10). However, for the oxidation mixtures, testing was only possible at a concentration of 10 µg/mL due to the low yield of the mixtures obtained.

As shown in Figure 10, all reference standards inhibited COX-2 expression compared to LPS treatment. Regarding the oxidation mixtures, those derived from rhamnetin exhibited a slight decrease in COX-2 expression compared to the control, with a slightly more pronounced inhibition at pH 2. On the other hand, the oxidation mixtures of quercetin also showed a mild reduction in enzyme expression, although less significant (see statistical analysis in the figure). The most notable difference was observed in the oxidation products of kaempferol: at pH 2, a statistically significant overexpression of COX-2 was detected, whereas at pH 7, a trend like that of quercetin was observed, with a slight decrease in enzyme levels.

These findings highlight the crucial role of flavonol metabolism in determining their biological effects. The metabolites generated during oxidation may either enhance or counteract COX-2 inhibition, demonstrating that these compounds do not always exert exclusively beneficial effects. In this case, the oxidation products of kaempferol at pH 2 and in an aprotic medium led to COX-2 overexpression, whereas inhibition was observed at pH 7 and in a protic medium. This suggests that subtle variations in pH and solvent can drastically influence biological activity.

The observed effects are likely due to multiple metabolites with potentially opposing activities, including BZFs and other phenolic derivatives identified in these mixtures. Despite this complexity, the inhibition of COX-2 observed in the oxidation products of rhamnetin at a concentration of 10 µg/mL suggests that certain metabolites may contribute to the beneficial effects of these mixtures. These results underscore the need for further studies to isolate and characterize the key metabolites involved in COX-2 regulation and to understand how metabolic transformations influence their pharmacological potential.

### 3.3. Computational Studies

#### 3.3.1. DFT Analysis

To gain a deeper understanding of the influence of electronic properties on the antioxidant capacity of flavonols and their oxidation products, two calculation methodologies based on density functional theory (DFT) were employed: the r2SCAN-D4/def2-TZVPP functional and the hybrid meta-GGA M06-2X functional. Key parameters were calculated to evaluate antioxidant capacity, including ionization potential (IP), hydrogen bond dissociation energy through the Wiberg bond index (BDEŋW), and chemical hardness (η) for flavonols A1–3, as well as for hydroxylated (BZFs-OH) and methoxylated (BZFs-OMe) benzofuranones present in their respective oxidation products (see Table 2) [51].

Ionization potential (IP) is a key descriptor for evaluating a compound’s ability to donate electrons in antioxidant mechanisms mediated by electron transfer (ET). As shown in Table 2, the IP values obtained with r2SCAN-D4/def2-TZVPP are systematically higher than those calculated with M06-2X and also show better agreement with the experimental values reported in the literature for flavonols such as quercetin and kaempferol (≈120 kcal/mol) [67,68,69,70].

From an antioxidant perspective, the IP values obtained with r2SCAN-D4/def2-TZVPP indicate that the flavonol precursors (quercetin, kaempferol, and rhamnetin) exhibit the lowest IP values, suggesting a greater ability to donate electrons compared to their oxidized BZF derivatives. Additionally, within the BZF derivatives, a clear trend is observed: methoxylated compounds (BZF-OMe) have lower IP values than their hydroxylated counterparts (BZF-OH). This suggests that methoxylation enhances electron donation, possibly through inductive and mesomeric effects, facilitating ionization and modulating the antioxidant activity of these compounds.

From an electronic reactivity perspective, the IP values obtained with r2SCAN-D4/def2-TZVPP exhibit better correlation with experimental data compared to M06-2X while also following a chemically consistent trend among structurally related compounds. In contrast, M06-2X displays a high degree of dispersion in the calculated values, with no clear trend among flavonols and their oxidized derivatives, which limits its predictive capability for evaluating antioxidant reactivity.

On the other hand, chemical hardness (η) is a key descriptor that quantifies a molecule’s resistance to changes in its electronic distribution, providing insights into its electronic stability and tendency to participate in redox processes. In general, molecules with lower hardness exhibit greater electronic flexibility, facilitating electron transfer and enhancing their antioxidant capacity.

As shown in Table 2, the η values calculated with M06-2X suggest that flavonols are significantly more complex than predicted by r2SCAN-D4/def2-TZVPP. However, both methods show the same general trend. Flavonols exhibit lower hardness than their BZF derivatives, aligning with their higher electronic reactivity and their experimentally observed antioxidant capacity.

From a more detailed perspective, the values obtained with r2SCAN-D4 show a trend more consistent with ionization potential (IP) values, highlighting that BZF-OMe derivatives have lower hardness than BZF-OH derivatives. This relationship is consistent with the lower ionization energy observed in BZF-OMe, suggesting that methoxylation reduces electronic hardness, facilitating electron donation and modifying the antioxidant reactivity of these compounds.

These findings support the reliability of r2SCAN-D4/def2-TZVPP in accurately describing the relationship between hardness, IP, and antioxidant activity, as it provides values that follow a chemically consistent pattern with the redox properties observed in flavonols and their oxidized derivatives.

The methodology proposed by Rohman et al. was employed to calculate BDE values [48]. This approach uses a model based on the enthalpy of dissociation of the O-H bond (BDE) with a simplified theoretical framework. This model provides precise and computationally efficient calculations compared to conventional quantum chemistry methods such as CCSD(T) and CBS-QB3. The BDE values obtained with M06-2X show better agreement with experimental data and literature reports (70–90 kcal/mol for the most reactive hydroxyl groups). This suggests that M06-2X is more suitable for describing phenoxide radicals’ stability and flavonols’ antioxidant capacity. In contrast, r2SCAN-D4/def2-TZVPP tends to overestimate radical stability, which could affect the correlation with experimental data from antioxidant assays (Table 3).

From a computational perspective, r2SCAN-D4/def2-TZVPP remains a viable option due to its lower computational cost, making it more suitable for high-throughput studies that require the evaluation of numerous compounds. Additionally, its IP and chemical hardness (η) values correlate better with experimental data, reflecting more consistent trends in flavonols’ electronic reactivity and oxidized derivatives.

On the other hand, although M06-2X is computationally more demanding in terms of time and resources, its BDE predictions exhibit better agreement with experimental values, particularly in assessing phenoxide radical stability. This suggests that M06-2X is more reliable for describing hydrogen atom transfer (HAT) mechanisms, whereas r2SCAN-D4/def2-TZVPP provides a more accurate description of electron transfer (ET) mechanisms.

Therefore, the choice of method depends on the study’s focus: r2SCAN-D4/def2-TZVPP is the preferred choice for evaluating redox properties and global trends in electronic reactivity. At the same time, M06-2X is more suitable when high precision in radical stability prediction and hydrogen abstraction processes is required.

Upon analyzing the obtained results, flavonols generally exhibit lower IP, BDEŋW, and chemical hardness (η) values than BZFs-OH and BZFs-OMe, which may be related to their higher antioxidant capacity observed in ORAC-FL and ORAC-PGR assays. Additionally, the ionization potentials of the three analyzed flavonols are higher than their respective BDEŋW values, suggesting that these compounds primarily act on radicals through the HAT mechanism. The O-H group with the lowest BDEŋW value corresponds to position 3 for all studied flavonols. The Fukui function consistently identifies this hydroxyl group as the most reactive site for radical interactions. This observation aligns with the proposed mechanism in Scheme 3 for the oxidation of flavonols to benzofuranones by AAPH, where the reaction begins with the abstraction of hydrogen atoms from these specific hydroxyl groups. The literature also supports this observation, as it has been reported that O-H groups at positions 3 or 4’ in flavonols such as quercetin and kaempferol play a central role in radical stabilization reactions [67,68,69,70].

In the case of BZF-OH, no significant differences were observed between the general electronic properties of BZF-OH and BZF-OMe in the r2SCAN-3c method. However, in the M06-2X method, BZF-OH exhibited lower BDE than BZF-OMe, especially BZF-OMe-kaempferol, which presented the highest BDE among all studied molecules. This result aligns well with those obtained in the ORAC-FL and ORAC-PGR assays, showing the lowest antioxidant capacity.

Additionally, like flavonols, the BDEŋW values are lower in energy than the IP values, suggesting that these compounds also primarily act through the HAT mechanism. Interestingly, in all BZF-OHs, the O-H group with the lowest BDEŋW value is at position 2 of ring C. However, the Fukui function indicates that the O-H groups at positions 3’ and 4’ are the most reactive centers toward radicals. This suggests versatility in the reactivity of BZF-OHs, where the most labile hydrogen is at position 2. In contrast, the highest radical reactivity is centered at positions 3’ and 4’, allowing these molecules to act as antioxidant agents through different positions (Table 3).

This finding is also important, as it aligns with previous observations. BZF-OMe lacks the O-H group at position 2 of ring C, which has the lowest BDEŋW value in oxidation products at neutral pH. This absence implies reduced versatility and a lower capacity to stabilize radicals. This could contribute to the observation that oxidation products obtained at pH 2 exhibit better antioxidant capacities in ORAC assays than those obtained at pH 7.

DFT calculations present various complexities, such as the choice of density functional, the basis function, and the simulation solvents, all of which can significantly affect the accuracy of the results [71]. Furthermore, as described above, oxidation products formed in situ during a reaction where an antioxidant stabilizes a radical also play an important role in the experimental measurement of antioxidant capacities. Even those metabolites formed under the same antioxidant and oxidizing conditions can vary in type and proportion, highlighting the depth and complexity of studying radical reactions, especially when they relate to a useful antioxidant effect at a physiological level. Despite these challenges, computational methods, such as DFT calculations, have great potential as predictors of reactivity. Particularly, for this study, they showed good agreement with experimental results and contributed significantly to the understanding of how different structural factors affect the reactivity and antioxidant capacity of compounds. However, it is important to note that DFT analyses are particularly useful for comparing molecules with significant structural differences, for example between flavonols and benzoic acid residues, but they are not very useful for comparing molecules with large structural similarities, since when having analogous structures, the electronic values are usually very close (less than 2 kcal/mol), which makes the results inconclusive at the level of the calculations usually used.

#### 3.3.2. Molecular Docking

On the other hand, taking into account that the oxidation products containing BZF-OH presented the highest antioxidant results, and that the great anti-inflammatory potential of BZF-OH has also been described as an inhibitor of the NF-kb pathway, we decided to carry out a docking and molecular dynamics study of these metabolites and their precursors, this time evaluating their anti-inflammatory perspective through the inhibition of the COX-2 enzyme [35,72].

**Figure 11:**
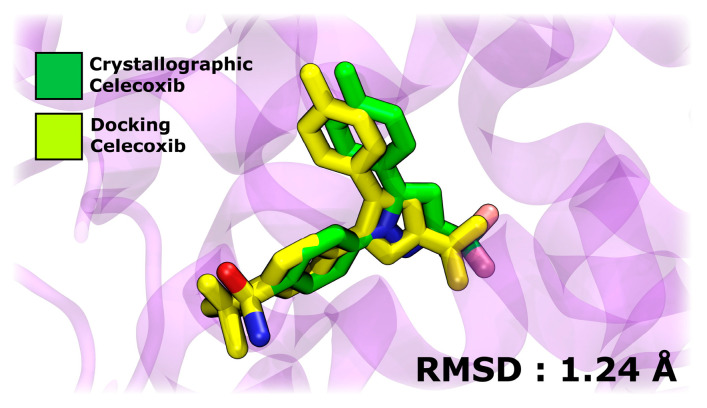
Structural superposition of crystallographic and docking-predicted conformations of celecoxib bound to COX-2.

In the initial stage of evaluating the interaction between A1, A2, A3, BZF-Quer-OH, BZF-Kaem-OH, and BZF-Ram-OH compounds and COX-2 receptors, molecular docking simulations were employed. The COX-2 monomer was used as the target protein, and celecoxib was included as a control to benchmark the docking results. The results obtained for the COX-2 complex with celecoxib show a high similarity between the conformations of the crystallographic structure of the ligand and those predicted by docking simulations, with a root mean square deviation (RMSD) of 1.24 Å. This low RMSD reflects the robustness of the molecular docking protocol, allowing for reliable testing of the compounds of interest in this study. Figure 11 presents a structural superposition highlighting the alignment between the experimental and simulated conformations.

The docking results and the corresponding binding scores, detailed in Table 4, reveal that celecoxib, used as a control, exhibits the most favorable binding affinity with a docking score of −10.5 kcal/mol. Among the analyzed compounds, BZF-Quer-OH shows the highest affinity with a score of −9.1 kcal/mol, followed by BZF-Kaem-OH (−8.9 kcal/mol), and quercetin (A1) (−8.9 kcal/mol). Kaempferol (A2) and rhamnetin (A3) display slightly lower affinities, with docking scores of −8.7 kcal/mol and −8.4 kcal/mol, respectively. BZF-Ram-OH demonstrates the lowest binding affinity among the evaluated compounds, with a docking score of −8.2 kcal/mol. The poses of these conformations were used as starting points for subsequent analysis via classical molecular dynamics simulations.

#### 3.3.3. COX-2/Ligand Molecular Dynamics and MM-GBSA Calculations

**Figure 12:**
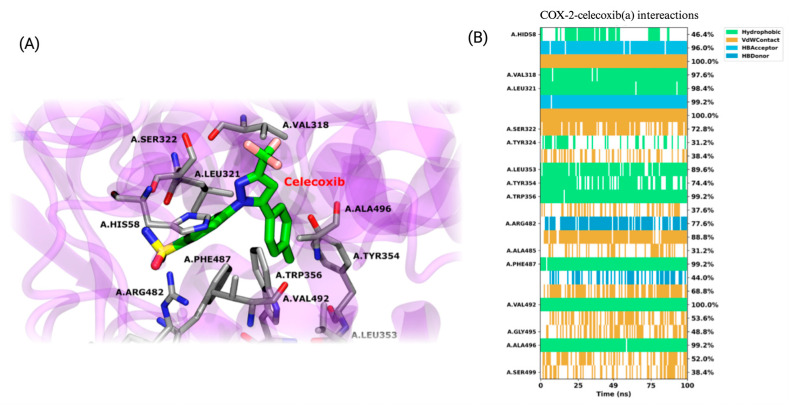
Structural analysis and interaction profile of the COX-2/celecoxib complex. (**A**) A zoomed-in view of the COX-2 active site, showing the conformation of celecoxib (monomer A) after 100 ns of molecular dynamics simulation. (**B**) Interaction types between the COX-2/celecoxib complex.

To evaluate the interaction between COX-2 and the compounds, classical all-atom molecular dynamics simulations were performed, using the docking results as the starting point for the structure of each complex. The results indicate a high level of stability for the crystallographic ligand celecoxib throughout the simulation. Celecoxib maintains key hydrogen bond interactions with residues HIS58, LEU321, ARG482, and PHE487 over the simulation. These hydrogen bonds play a crucial role in stabilizing the ligand in the active site, with ARG482 and PHE487 showing consistent hydrogen bonding over a significant portion of the simulation time, as represented by the blue lines in Figure 12. In addition, the ligand exhibits hydrophobic interactions with several important residues, such as VAL318, LEU321, TRP356, VAL492, and ALA496. These hydrophobic interactions, highlighted in green, are stable over the 100 ns of simulation. Van der Waals (VdW) interactions, shown in orange, are also relevant in stabilizing the complex, with frequent interactions observed with HIS58, SER322, ARG482, PHE487, and ALA485. Although the complex reaches a stable conformation, the natural side-chain motions of certain residues cause fluctuations in specific interactions, reflecting the inherent dynamics of molecular systems even at equilibrium.

**Figure 13:**
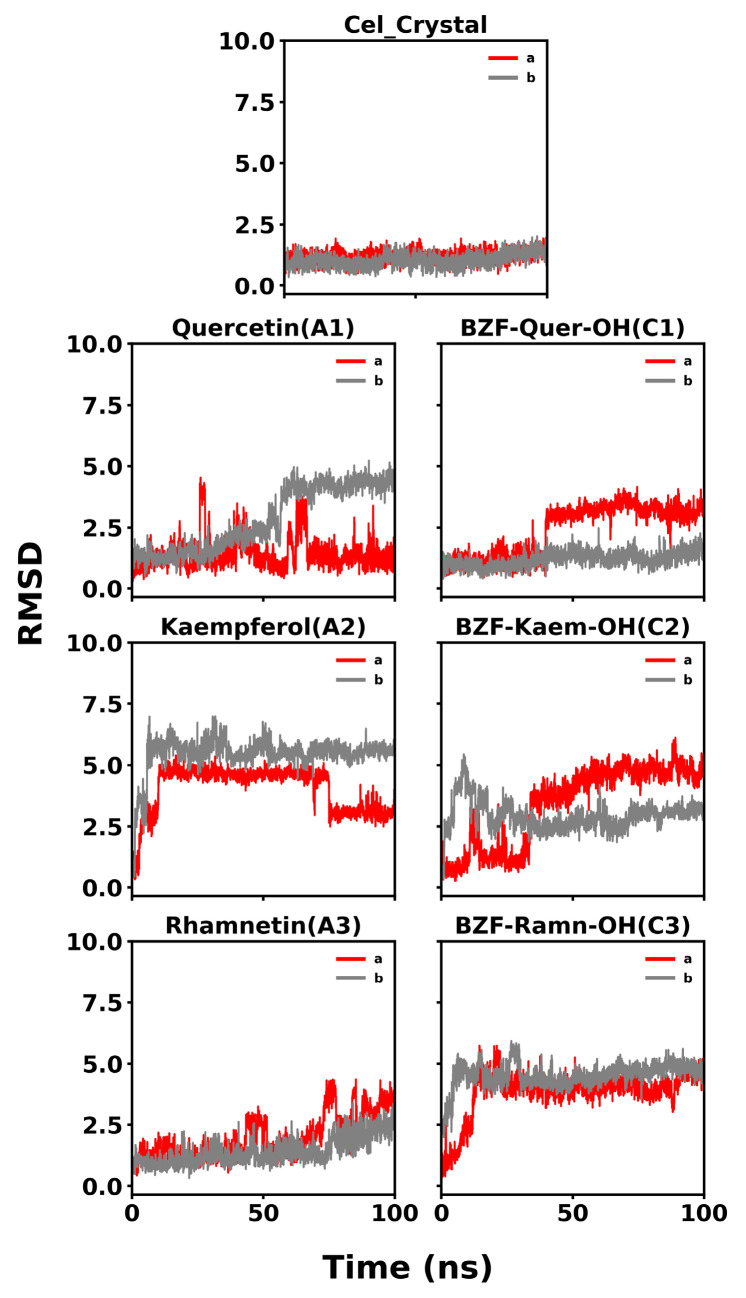
RMSD profiles of COX-2/ligand complexes over 100 ns of molecular dynamics simulations. (a) RMSD profiles for COX-2 complexes with natural ligands: Quercetin (A1), Kaempferol (A2), Rhamnetin (A3), and the crystallographic reference Celecoxib. (b) RMSD profiles for COX-2 complexes with BZF-OH derivatives: BZF-Quer-OH (C1), BZF-Kaem-OH (C2), and BZF-Ramn-OH (C3). The red line corresponds to the ligand RMSD and the gray line to the protein backbone.

The RMSD plots, detailed in Figure 13, show the stability of the COX-2 dimer complexes with celecoxib and the analyzed compounds over 100 ns of molecular dynamics simulations. For celecoxib, the RMSD remains consistently low, around 2.0 Å for both monomers, indicating a stable interaction and strong agreement with the initial docking pose. This confirms the accuracy and reliability of the docking protocol for celecoxib and demonstrates that the molecular dynamics simulations could reproduce the crystallographic ligand pose over the simulation time.

In the case of quercetin (A1), the RMSD initially fluctuates but reaches a plateau around 3.0–4.0 Å after about 50 ns. Although the RMSD is higher than that of the control, the system achieves a relatively stable conformation during the second half of the simulation, suggesting that the initial docking pose may have adjusted but eventually stabilized. BZF-Quer-OH (C1) also shows some initial fluctuations, but it stabilizes into a plateau around 4.0 Å after approximately 40 ns. This indicates that the complex undergoes rearrangement from the initial docking pose but reaches a stable conformation as the simulation progresses.

For kaempferol (A2), the RMSD exhibits more substantial fluctuations between 3.0 and 6.0 Å but is stable in the active site, which is more evident in one of the monomers (b). The high RMSD values suggest significant deviation from the initial docking pose, indicating that the docking pose was further from the conformation stabilized by the molecular dynamics simulation. Similarly, BZF-Kaem-OH (C2) fluctuates between 3.0 and 6.0 Å, though it appears to begin stabilizing around 2.5–5.0 Å toward the end of the simulation.

Finally, rhamnetin (A3) shows an increasing trend in RMSD, reaching a plateau around 2.5–3.0 Å after 60 ns, suggesting it achieves a stable conformation after initial fluctuations. In the case of BZF-Ramn-OH (C3), the RMSD stabilizes around 5.0 Å, indicating that the complex undergoes some structural rearrangements early in the simulation but eventually settles into a more stable conformation.

**Figure 14:**
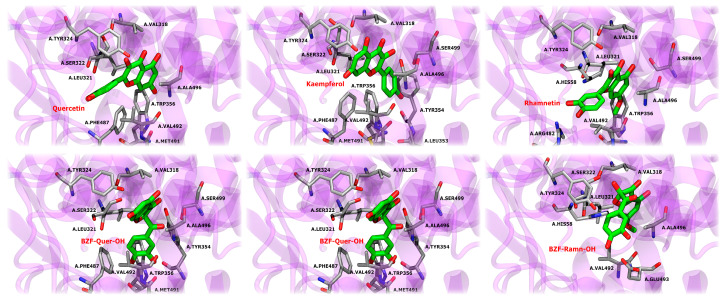
RMSD profiles of COX-2/ligand complexes over 100 ns of molecular dynamics simulations.

The stability of the compounds within the active site is governed by various types of interactions between the ligands and the active site residues, some of which are similar to those observed with the crystallographic ligand celecoxib. These interactions are described in Figure 14 and Figures S39 and S40. The interaction profiles of six ligands across the two COX-2 monomers reveal several common patterns that contribute to their stabilization within the active site and unique features that differentiate their binding modes.

In detail, hydrophobic interactions are a dominant force across all ligands, particularly with residues LEU321, PHE487, and VAL492, which consistently show high interaction frequencies in both monomers. These residues are critical for the stable positioning of the ligands within the active site, mirroring similar interactions observed with the crystallographic ligand celecoxib. The ALA496 and VAL318 residues also contribute to ligand stabilization through hydrophobic interactions, although their involvement varies slightly between ligands and monomers. In turn, van der Waals (VdW) interactions play a complementary role in ligand stabilization. Notably, SER322 and TYR324 are frequently involved in contact with most ligands, contributing to their consistent positioning within the binding pocket. These residues are particularly important in maintaining a stable interaction across both monomers, with SER322 often acting as a contact point in most ligands, including those that interact with celecoxib. Hydrogen bonding is essential in stabilization, with MET491 and ARG482 frequently establishing hydrogen bonds, especially in specific monomers. MET491, in particular, is critical for stabilizing several ligands, including BZF-Quer-OH, which exhibits frequent hydrogen bonding with this residue in monomer B. SER499 also engages in hydrogen bonds in multiple ligands, although less consistently.

Some ligands exhibit unique binding characteristics. Kaempferol and its derivative BZF-Kaem-OH show strong pi-stacking interactions with PHE487 in monomer B, a feature that sets them apart from the other ligands. This π-stacking interaction adds a distinctive mode of stabilization that is not observed as frequently in the other ligands. BZF-Quer-OH demonstrates increased hydrogen bonding with MET491 and HIS58 in monomer B, indicating a stronger reliance on hydrogen bonds in that monomer than other ligands. Similarly, rhamnetin displays frequent van der Waals interactions with TYR354 and SER322 in monomer A, contributing to its unique binding profile.

When compared to celecoxib, the ligands share several key interaction features. Hydrophobic contacts with LEU321, PHE487, and VAL492 are central to the binding of celecoxib and are also critical for the six ligands studied here. These residues provide a stable hydrophobic environment, ensuring the ligands remain tightly bound within the COX-2 active site. Additionally, SER322 and TYR324 exhibit van der Waals interactions in celecoxib and the ligands, reinforcing the importance of these residues as common interaction points in COX-2’s active site. Hydrogen bonding with MET491, a key interaction in celecoxib’s binding, is also frequently observed in the ligands, although the strength and consistency of these bonds vary between ligands.

The MM-GBSA interaction energy analysis reveals that celecoxib exhibits the strongest binding affinity to COX-2, with values of −48.4 kcal/mol and −46.6 kcal/mol for monomers (a) and (b), respectively, confirming its stability and strong interaction profile. Among the analyzed compounds, BZF-Ram-OH stands out with interaction energies close to celecoxib in monomer (a) (−44.7 kcal/mol). However, it shows reduced binding in monomer (b) (−30.9 kcal/mol), indicating variability in stability. Rhamnetin (A3) also shows relatively strong binding, with consistent interaction energies around −31.9 kcal/mol. BZF-Quer-OH and BZF-Kaem-OH display moderate binding affinities, with slightly stronger interactions in one monomer than the other. Kaempferol (A2) and quercetin (A1) exhibit the weakest interactions, with quercetin showing the least favorable energies, which correlates with its more fluctuating and less stable binding observed in the RMSD analysis. The details of these energies can be found in Table 5.

The results from COX-2 expression assays and molecular docking studies suggest that flavonol oxidation products modulate inflammatory activity in diverse ways. While oxidation mixtures derived from rhamnetin exhibited moderate COX-2 inhibition, those obtained from kaempferol at pH 2 unexpectedly induced significant enzyme overexpression. This indicates that oxidative transformations of flavonols can generate metabolites with opposing effects on inflammation, depending on the chemical environment.

Computational studies further support the hypothesis that benzofuranones (BZFs) are primarily responsible for the inhibition observed in biological assays. Specifically, BZF-Quer-OH exhibited a COX-2 binding affinity (−9.1 kcal/mol) comparable to the selective inhibitor celecoxib (−10.5 kcal/mol), suggesting a potential competitive inhibition mechanism. However, the inhibition observed in cellular assays was more moderate, indicating that other metabolites in the oxidation mixtures might limit its effect. Some compounds could interfere with BZF inhibitory activity or influence their stability and bioavailability in biological systems.

The simulation results reinforce this hypothesis, demonstrating that BZF-OH derivatives have a high affinity for COX-2 and form stable complexes within the enzyme’s active site. This structural stability, combined with their remarkable antioxidant properties, suggests that BZF-OH compounds may exert a dual protective effect against oxidative stress and inflammation. Additionally, the influence of pH and reaction medium on metabolite formation highlights the importance of optimizing conditions to enhance their biological activity.

These findings pave the way for further investigations to evaluate the behavior of BZF-OH compounds in biological models, enabling validation of their therapeutic potential and elucidation of their mechanisms of action. Since their effects may depend on interactions with other oxidation-derived metabolites, future research should focus on characterizing these compounds individually and assessing their impact on COX-2 modulation. In this context, BZF-OH emerges as a promising candidate for the development of multifunctional therapies targeting inflammatory and oxidative stress-related diseases.

## 4. Conclusions

This study demonstrates that the controlled oxidation of flavonols produces metabolites with distinct antioxidant and anti-inflammatory properties that are significantly influenced by pH, oxidant type, and solvent polarity. Products generated at pH 2 in a polar aprotic medium exhibited higher antioxidant activity in ORAC-FL, ORAC-PGR, and CAA assays than those obtained at pH 7 in a polar protic medium. Among them, rhamnetin oxidation products (Mox-Ram pH2) showed the highest antioxidant capacity and moderate COX-2 inhibition, suggesting therapeutic potential. Conversely, kaempferol oxidation products at pH 2 induced COX-2 overexpression, underscoring the complexity of their biological effects and the need to fine-tune oxidation conditions to maximize efficacy.

To better assess the impact of oxidation on antioxidant activity, the gross antioxidant capacity (GAC) concept was introduced. This approach revealed that oxidation products significantly contribute to ORAC assay results, redefining how antioxidant capacity should be interpreted in flavonol studies.

From a computational perspective, DFT calculations identified key electronic descriptors (IP and BDE_n_W) that correlated strongly with ORAC assay results based on the HAT mechanism. Additionally, molecular docking and dynamics simulations predicted high COX-2 affinity for BZFs, particularly BZF-Quer-OH, which exhibited binding affinities comparable to celecoxib, a known selective inhibitor. These predictions were validated by in vitro assays, reinforcing computational modeling as a valuable predictive tool for bioactive compound development. However, the lack of correlation between GAC and COX-2 affinity suggests that antioxidant and anti-inflammatory activities operate through independent mechanisms.

Overall, these findings confirm that oxidation products with enhanced bioactivity can be designed to validate the integration of experimental and computational approaches as a robust strategy for developing novel therapies against oxidative stress and chronic inflammation. Future research should focus on the individual characterization of key metabolites, such as BZF-OH, to validate their biological applicability and explore their potential as dual therapeutic agents.

## Figures and Tables

**Table 1:** ORAC-FL and ORAC-PGR indices for the samples studied.

Sample	ORAC Index		CAA (%)	Viability (%)		
	PGR	FL		HFF (10 µg/mL)	RAW 264.7	
					(10 µg/mL)	(100 µg/mL)
Quercetin (A1)	2.32 ± 0.20 ****	12.43 ± 0.37 ****	87.8 ± 0.4 ****	100 ± 14 *	98 ± 5	89 ± 4
Mox-Quer pH2 (C1)	0.88 ± 0.01 ***	8.98 ± 1.14 ****	91.2 ± 0.4 **	100 ± 7 *	100 ± 3	Nd ^1^
Mox-Quer pH7 (D1)	Low reactive	5.43 ± 0.07 ****	55.3 ± 1.5 ****	100 ± 12	100 ± 2	Nd ^1^
Kaempferol (A2)	3.19 ± 0.18 ****	11.05 ± 0.19 ****	68.9 ± 1.1 ****	100 ± 6	100 ± 5	98 ± 3
Mox-Kam pH2 (C2)	0.49 ± 0.08 **	6.80 ± 0.43 ****	93.3 ± 2.3 *	100 ± 3	95 ± 4	Nd ^1^
Mox-Kam pH7 (D2)	0.46 ± 0.03 **	4.77 ± 0.13 ****	76.1 ± 1.1 ****	89 ± 6	100 ± 8 *	Nd ^1^
Rhamnetin (A3)	3.25 ± 0.16 ****	5.02 ± 0.17 ****	53.2 ± 2.9 ****	70 ± 7 ****	100 ± 3	93 ± 4
Mox-Ram pH2 (C3)	1.04 ± 0.09 ****	6.63 ± 0.37 ****	81.0 ± 2.5 ****	88 ± 3	100 ±5	Nd ^1^
Mox-Ram pH7 (D3)	Low reactive	5.23 ± 0.17 ****	54.5 ± 3.9 ****	85 ± 3	98 ± 2	Nd ^1^
Trolox	1.00 ± 0.02	1.00 ± 0.05	Nd ^1^	Nd ^1^	Nd ^1^	Nd ^1^
Luteolin	Nd ^1^	5.11 ± 0.45 ****	Nd ^1^	Nd ^1^	Nd ^1^	Nd ^1^
Rosmarinic acid	Nd ^1^	4.31 ± 0.28 ****	Nd ^1^	Nd ^1^	Nd ^1^	Nd ^1^
Ferulic acid	Nd ^1^	2.84 ± 0.36 ***	Nd ^1^	Nd ^1^	Nd ^1^	Nd ^1^

^1^ Not determined. One-way ANOVA with Dunnett’s post-test, * *p* < 0.05, ** *p* < 0.01, *** *p* < 0.001, and **** *p* < 0.0001 indicate statistically significant differences concerning Trolox. CAA and viability were determined in HFF cells at 10 µg/mL, while viability assays were performed in HFF and RAW 264.7 cells. HFF cells were tested at 10 µg/mL, and RAW 264.7 cells were tested at 10 and 100 µg/mL. ORAC-FL (oxygen radical absorbance capacity with fluorescein), ORAC-PGR (oxygen radical absorbance capacity with pyrogallol red), CAA (cellular antioxidant activity), and HFF (human foreskin fibroblasts). RAW 264.7 is a murine macrophage cell line derived from BALB/c mice transformed with the Abelson murine leukemia virus (Ab-MuLV) and is widely used to study inflammation and immune responses.

**Table 2:** Global electronic properties in kcal mol ^−1^.

η (r2SCAN-D4/def2-TZVPP/M06-2X)	IP (r2SCAN-D4/def2-TZVPP/M06-2X)	Molecule
89.8/122.1	123.2/91.0	Quercetin
90.2/124.9	124.0/99.5	Kaempferol
89.7/119.9	122.7/89.5	Rhamnetin
103.6/149.3	141.0/56.2	Quer-BZF-OH
106.1/179.3	146.4/117.5	Kaem-BZF-OH
102.5/151.6	140.8/60.8	Ram-BZF-OH
100.7/145.0	138.8/94.9	Quer-BZF-OMe
102.9/203.7	143.4/215.7	Kam-BZF-OMe

**Table 3:** Values of radical Fukui function and BDEŋW.

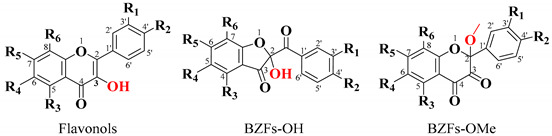			
BDE_ηW_ (r2SCAN-D4/def2-TZVPP/M06-2X)	$f_{k}^{0}$ (r2SCAN-D4/def2-TZVPP/M06-2X)		
58.39/79.38	0.058/0.064	3-OH	Quercetin
61.98/84.26	0.026/0.016	5-OH	
61.98/84.26	0.026/0.018	7-OH	
61.98/84.26	0.029/0.022	3’-OH	
61.08/83.04	0.042/0.039	4’-OH	
58.61/80.71	0.062/0.070	3-OH	Kaempferol
62.22/85.68	0.029/0.017	5-OH	
62.22/85.68	0.028/0.018	7-OH	
62.22/85.68	0.042/0.036	4’-OH	
58.28/77.90	0.057/0.065	3-OH	Rhamnetin
61.87/82.69	0.028/0.017	5-OH	
61.87/82.69	0.028/0.021	3’-OH	
60.97/81.49	0.041/0.037	4’-OH	
67.34/97.07	0.022/0.019	2-OH	BZF-OH-Quercetin
70.44/101.55	0.028/0.004	4-OH	
71.48/103.05	0.015/0.005	6-OH	
70.44/101.55	0.059/0.86	3’-OH	
69.41/100.06	0.055/0.069	4’-OH	
68.97/115.89	0.025/0.023	2-OH	BZF-OH-Kaempferol
72.16/121.25	0.042/0.056	4-OH	
73.22/123.03	0.019/0.025	6-OH	
73.22/123.03	0.054/0.021	4’-OH	
66.64/98.52	0.023/0.019	2-OH	BZF-OH-Rhamnetin
69.71/103.06	0.031/0.004	4-OH	
69.71/103.06	0.055/0.086	3’-OH	
68.69/101.55	0.052/0.069	4’-OH	
68.50/98.62	0.026/0.004	4-OH	BZF-OMe-Quercetin
69.51/100.07	0.019/0.005	6-OH	
68.50/98.62	0.060/0.084	3’-OH	
68.50/98.62	0.053/0.069	4’-OH	
69.97/138.52	0.035/0.056	4-OH	BZF-OMe-Kaempferol
71.00/140.56	0.023/0.026	6-OH	
71.00/140.56	0.046/0.020	4’-OH	

**Table 4:** Docking scores of celecoxib and analyzed compounds in interactions with COX-2 using AutoDock-GPU.

Docking Score (kcal/mol)	Molecule
−10.5	Celecoxib
−8.9	Quercetin (A1)
−8.7	Kaempferol (A2)
−8.4	Rhamnetin (A3)
−9.1	BZF-Quer-OH
−8.9	BZF-Kaem-OH
−8.2	BZF-Ram-OH

**Table 5:** Interaction energies (MM-GBSA) of celecoxib and analyzed compounds with COX-2 in both monomers (a) and (b).

MM-GBSA(b) (kcal/mol)	MM-GBSA(a) (kcal/mol)	Molecule
−46.6	−48.4	Celecoxib
−27.7	−28.2	Quercetin (A1)
−29.7	−29.9	Kaempferol (A2)
−31.8	−31.9	Rhamnetin (A3)
−30.2	−31.9	BZF-Quer-OH
−28.3	−32.2	BZF-Kaem-OH
−30.9	−44.7	BZF-Ram-OH

## Data Availability

Data is contained within the article and Supplementary Materials.

## Supplementary Materials

The following supporting information can be downloaded at: https://www.mdpi.com/article/10.3390/antiox14040479/s1.
