# Supplementary material for: Gross Antioxidant Capacity and Anti-Inflammatory Potential of Flavonol Oxidation Products: A Combined Experimental and Theoretical Study
Source: Antioxidants (Basel). 2025 Apr 16;14(4):479. doi: 10.3390/antiox14040479 (PMC12024297; doi:10.3390/antiox14040479)
Supplement: Supplementary file 1 [file antioxidants-14-00479-s001.zip › antioxidants-3576477-supplementary.pdf]

Gross Antioxidant Capacity and Anti-Inflammatory Potential of Flavonol Oxidation Products: A Combined Experimental and Theoretical Study: Supplementary information

Karen Acosta-Quiroga [1,2*], Esteban Rocha-Valderrama [1,2], Matías Zúñiga-Bustos [2], Raúl Mera-Adasme [3], Gustavo Cabrera-Barjas [4], Claudio Olea-Azar [1], Mauricio Moncada-Basualto [2*]

[1] Laboratorio de Radicales Libres y Antioxidantes, Facultad de Ciencias Químicas y Farmacéuticas, Universidad de Chile
[2] Instituto Universitario de Investigación y Desarrollo Tecnológico, Universidad Tecnológica Metropolitana.
[3] Departamento de Química, Facultad de Ciencias, Universidad de Tarapacá.
[4] Facultad de Ciencias para el Cuidado de la Salud, Universidad San Sebastián, Campus Las Tres Pascualas, Lientur 1457, Concepción 4080871, Chile
* Correspondence: mmoncadab@utem.cl, karenacosta@ug.uchile.cl; Tel.: (optional; include country code; if there are multiple corresponding authors, add author initials)

1. Chemical Section

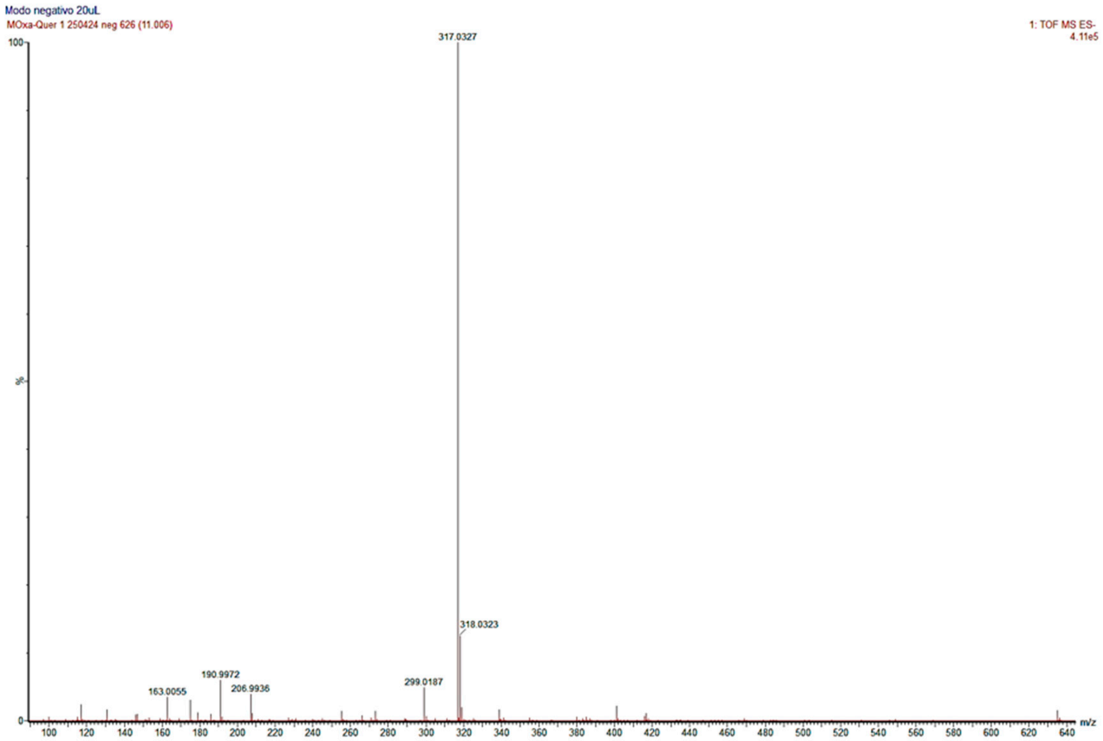

Figure S1. Mass spectrum of peak 2 from C1 MOx.

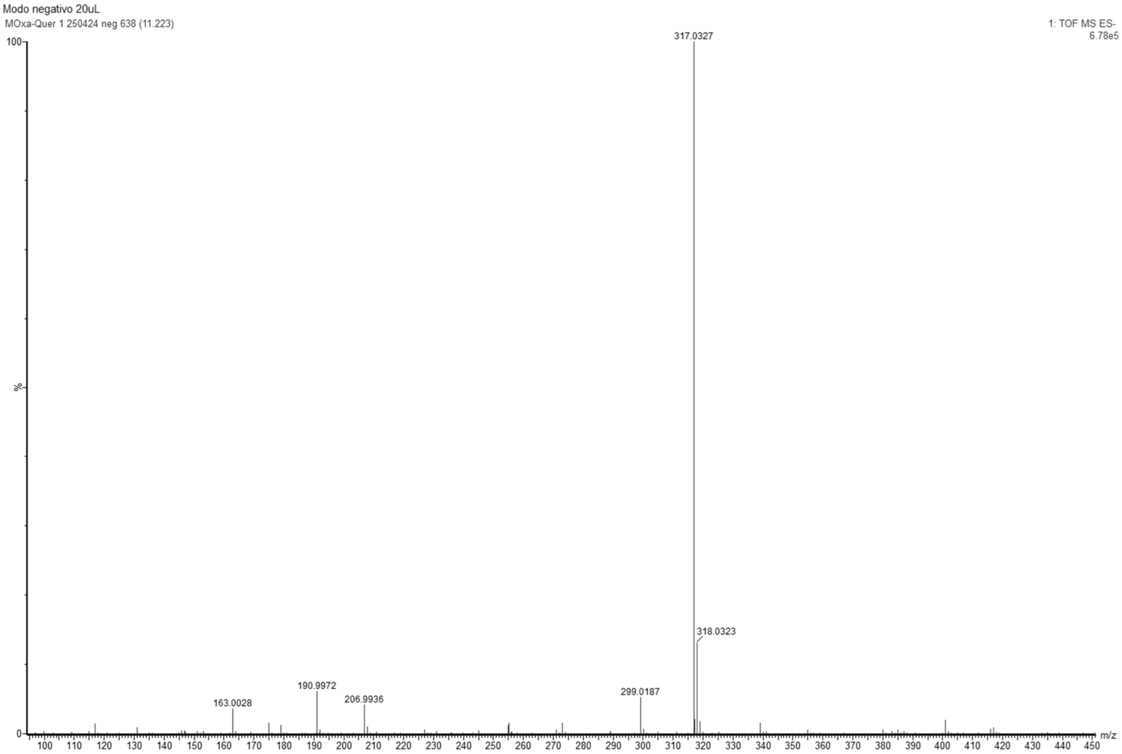

Figure S2. Mass spectrum of peak 3 from C1 MOx.

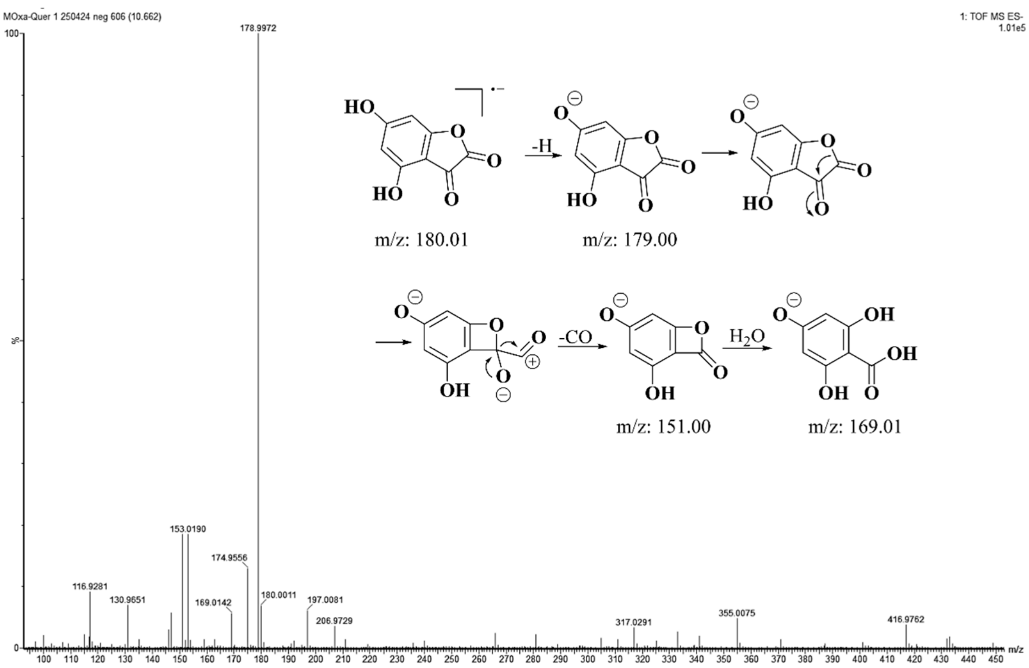

Figure S3. Mass spectrum of peak 1 from C1 MOx.

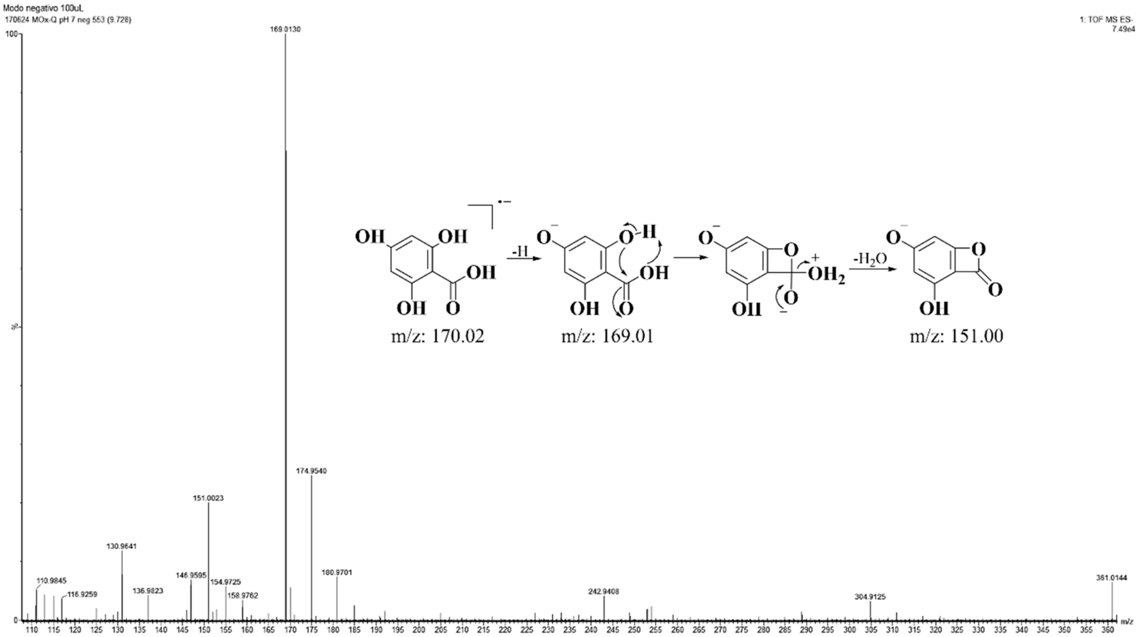

Figure S4. Mass spectrum of peak 1 from D1 MOx

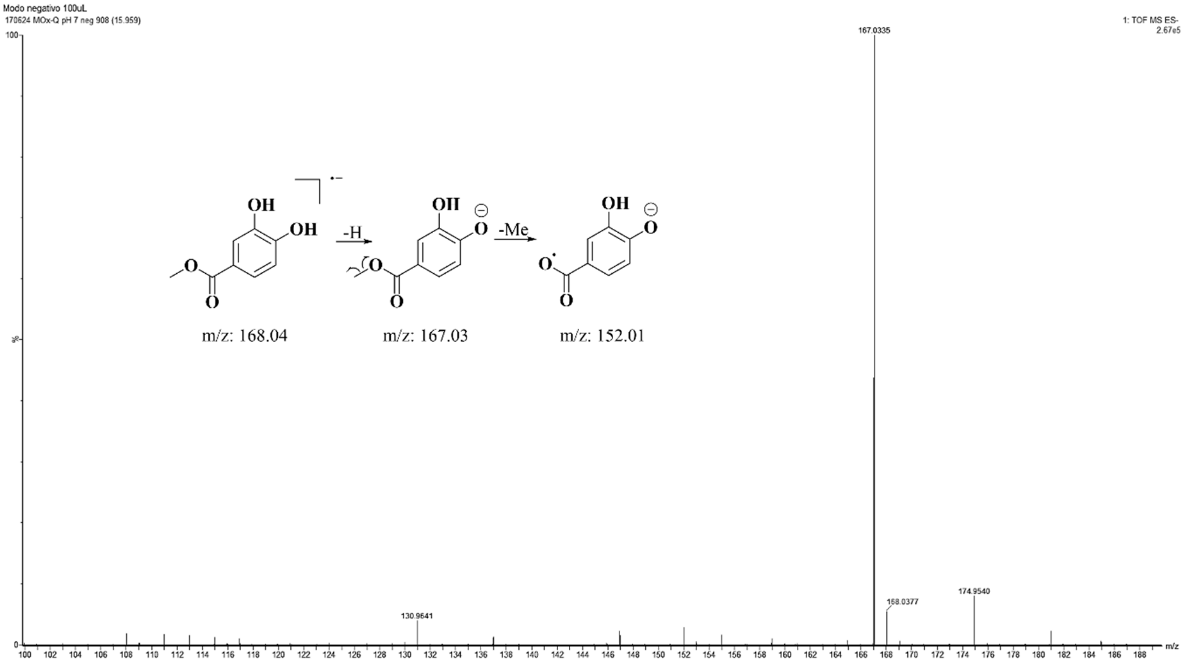

Figure S5. Mass spectrum of peak 2 from D1 MOx.

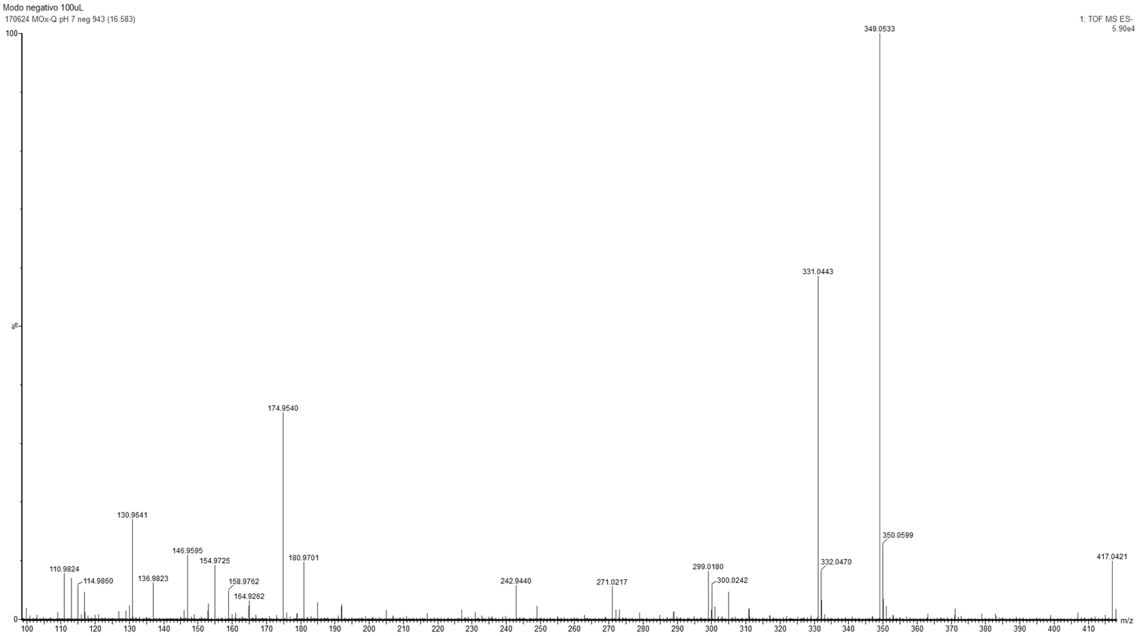

Figure S6. Mass spectrum of peak 3 from D1 MOx.

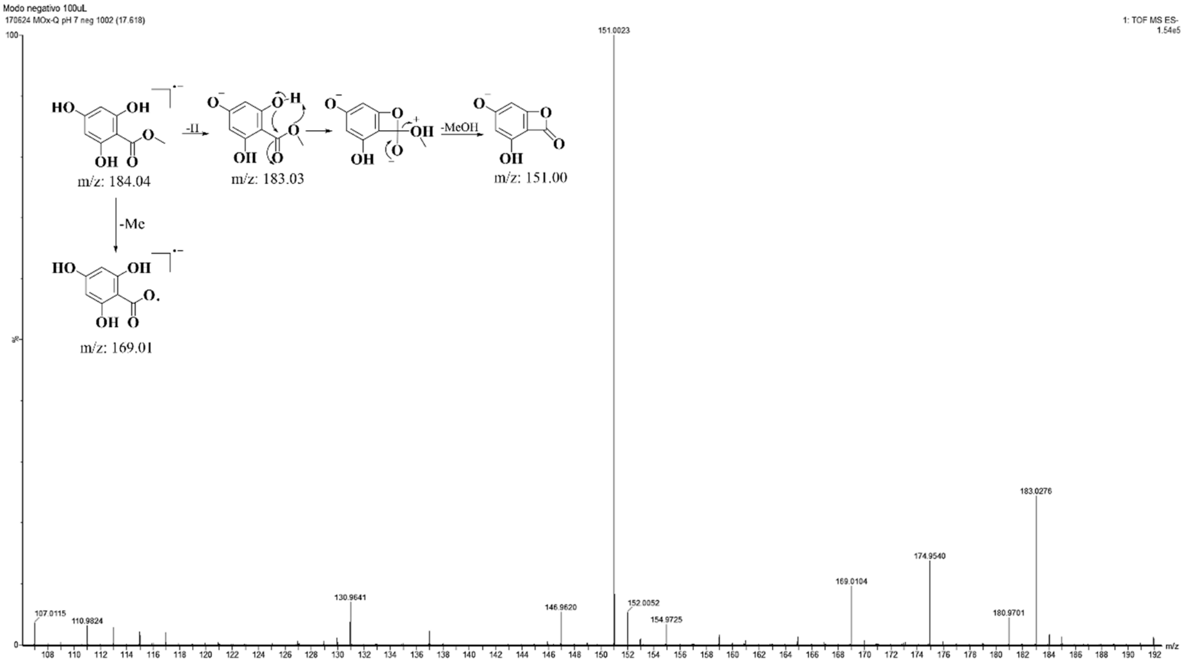

Figure S7. Mass spectrum of peak 4 from D1 MOx.

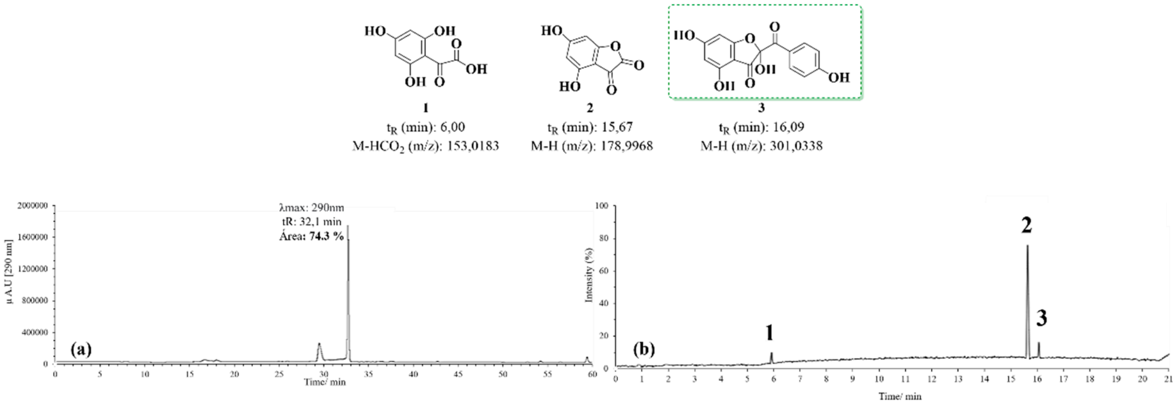

Figure S8. Chromatograms of kaempferol MOx at pH 2 (C2): (a) HPLC-DAD, (b) UPLC-Q-TOF.

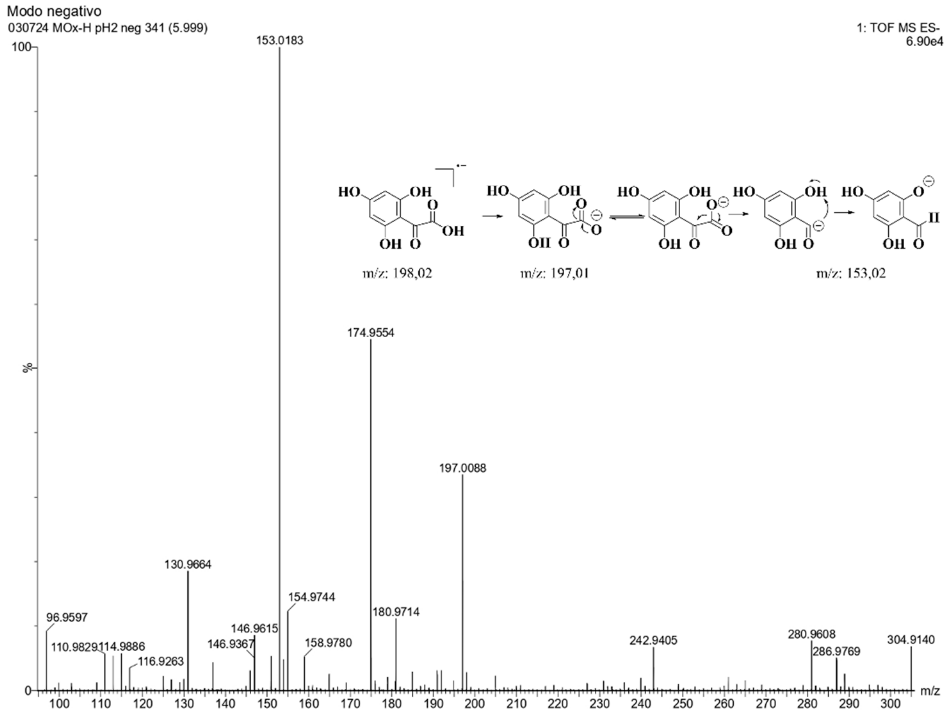

Figure S9. Mass spectrum of peak 1 from C2 MOx.

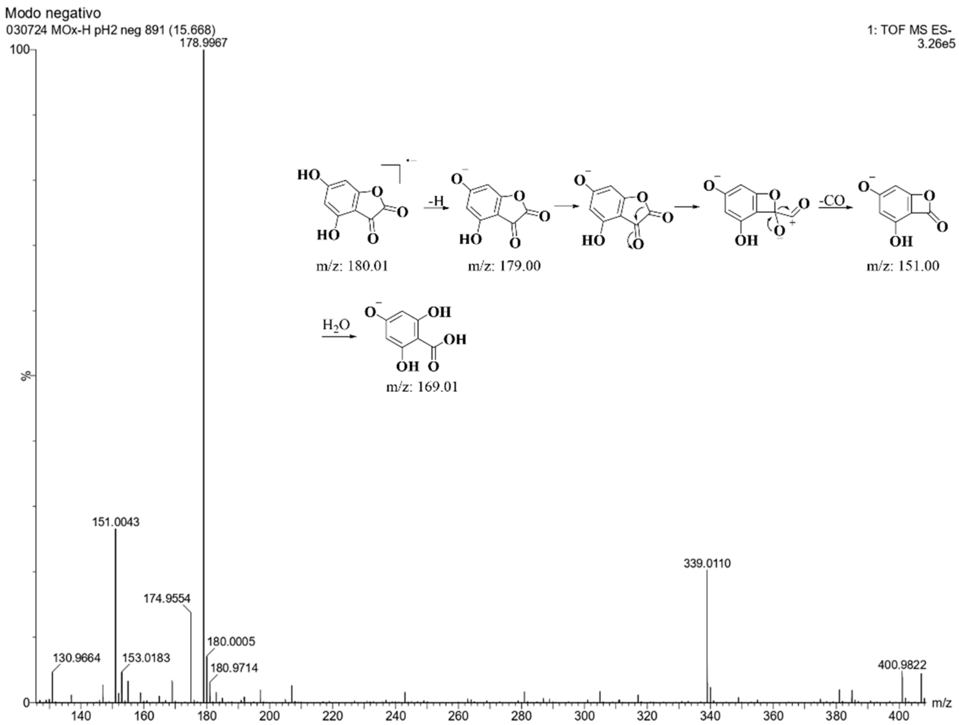

Figure S10. Mass spectrum of peak 2 from C2 MOx

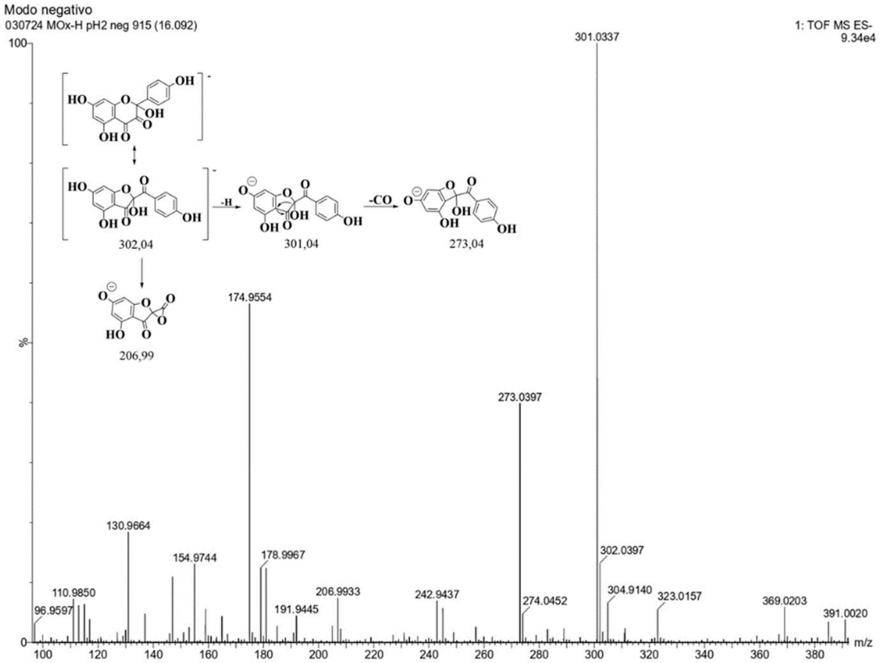

Figure S11. Mass spectrum of peak 3 from C2 MOx.

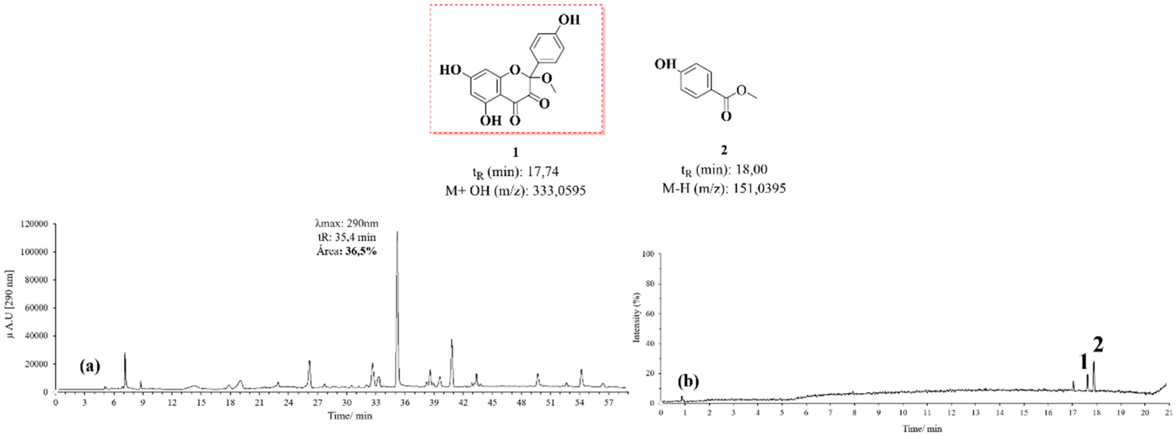

Figure S12. Chromatograms of kaempferol MOx at pH 7 (D2): (a) HPLC-DAD, (b) UPLC-Q-TOF.

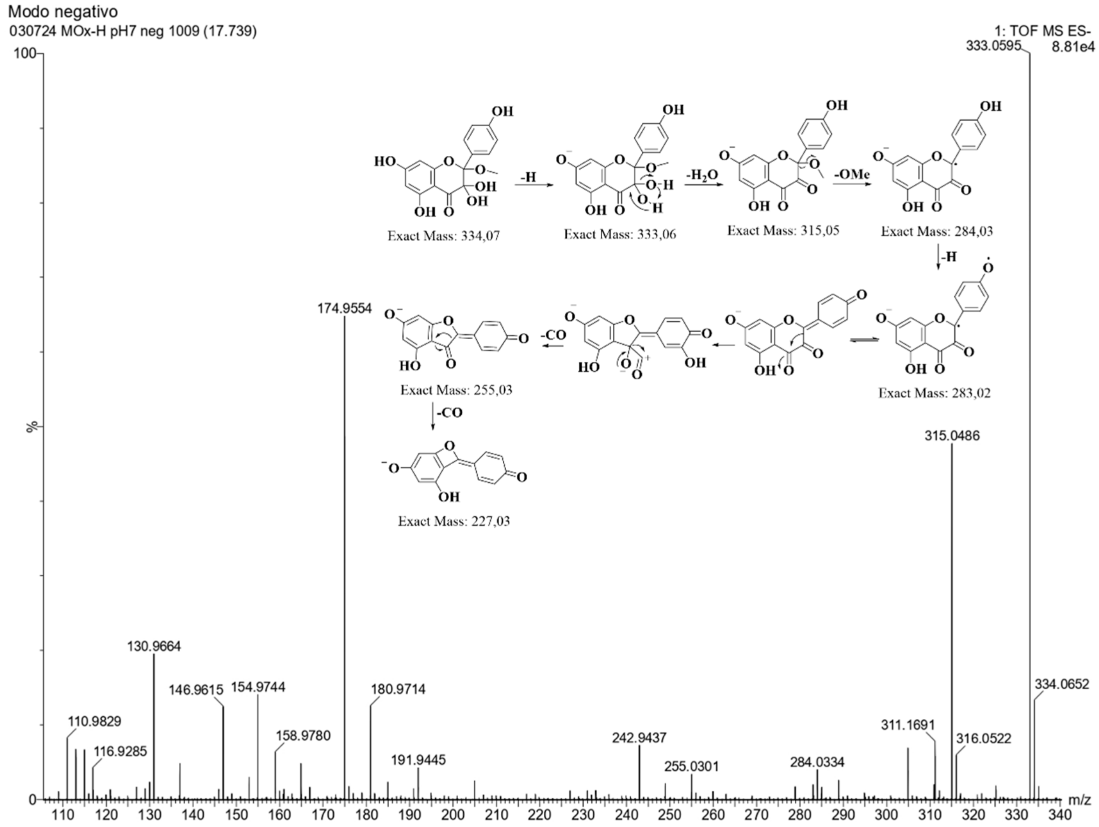

Figure S13. Mass spectrum of peak 1 from D2 MOx

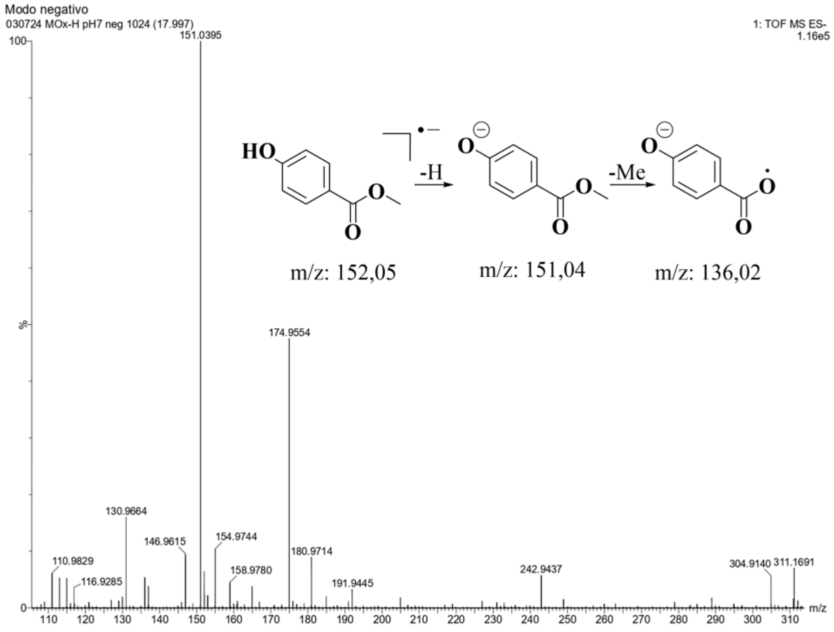

Figure S14. Mass spectrum of peak 2 from C2 MOx.

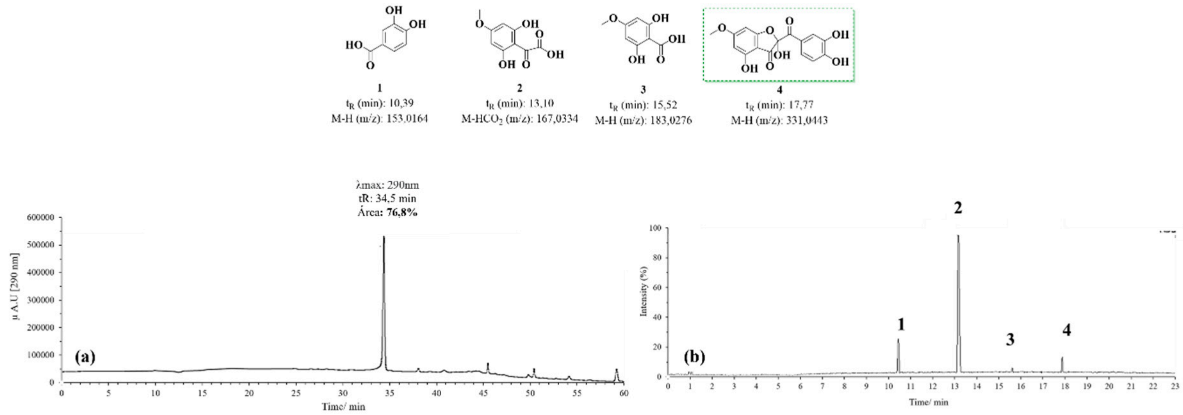

Figure S15. Chromatograms of rhamnetin MOx at pH 2 (C3): (a) HPLC-DAD, (b) UPLC-Q-TOF.

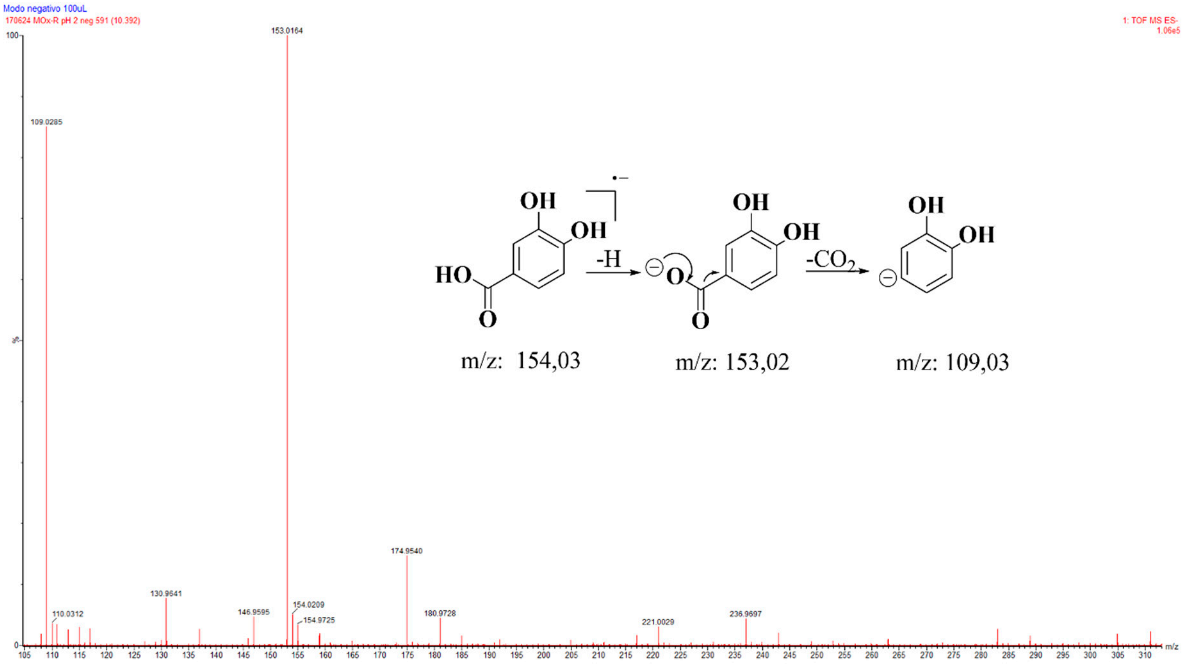

Figure S16. Mass spectrum of peak 1 from C3 MOx.

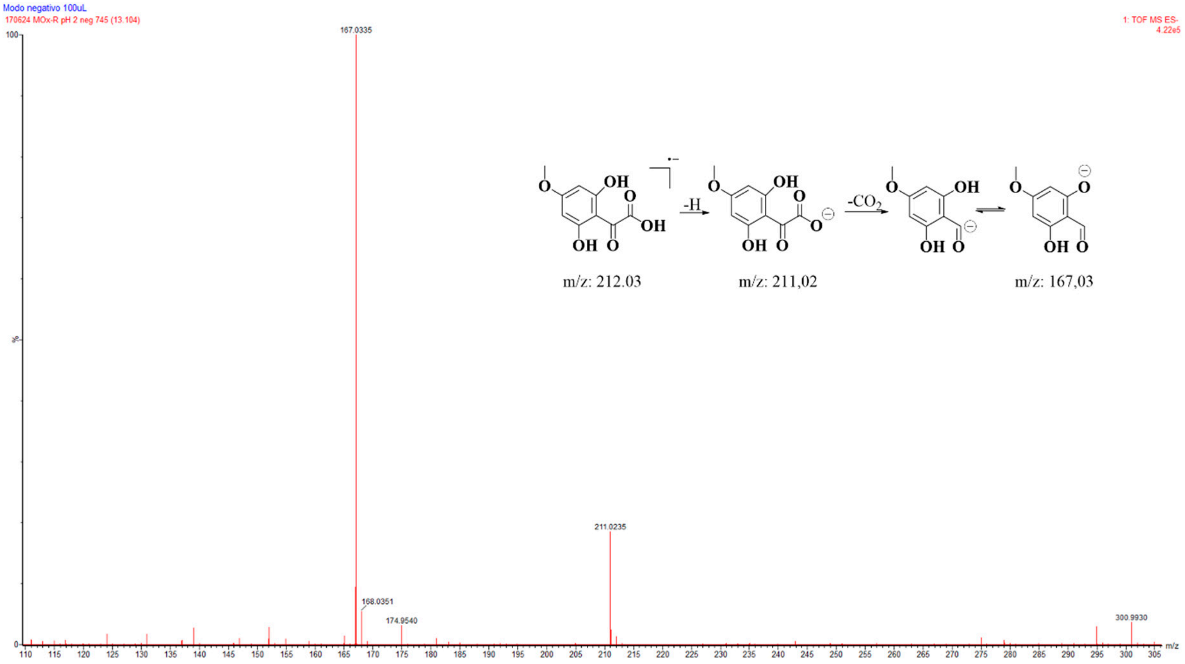

Figure S17. Mass spectrum of peak 2 from C3 MOx.

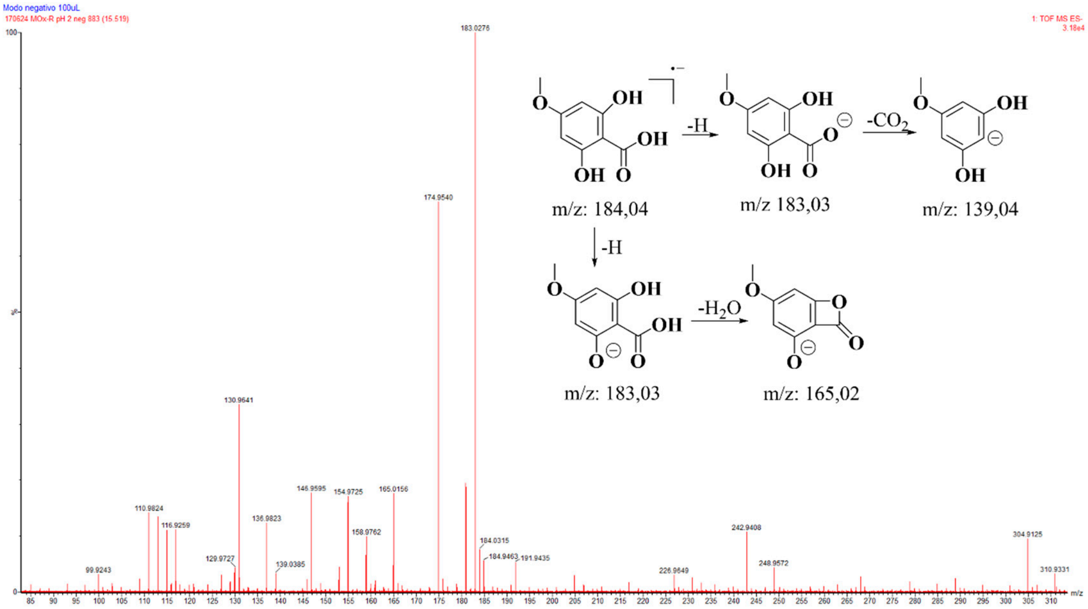

Figure S18. Mass spectrum of peak 3 from C3 Mox.

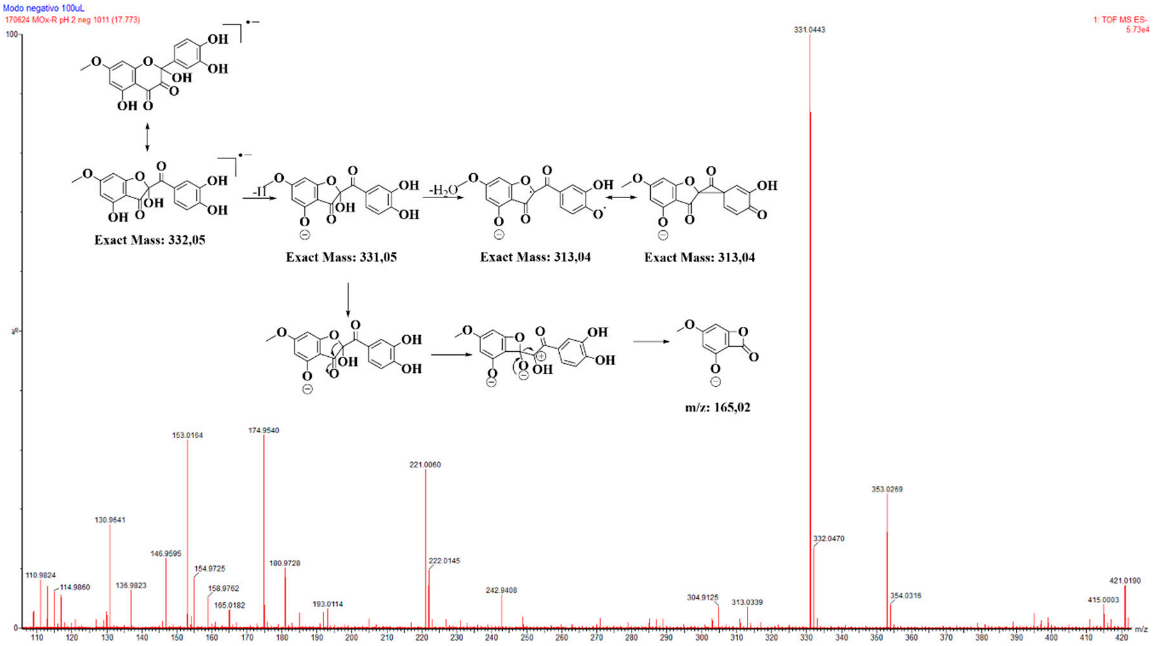

Figure S19. Mass spectrum of peak 4 from C3 MOx.

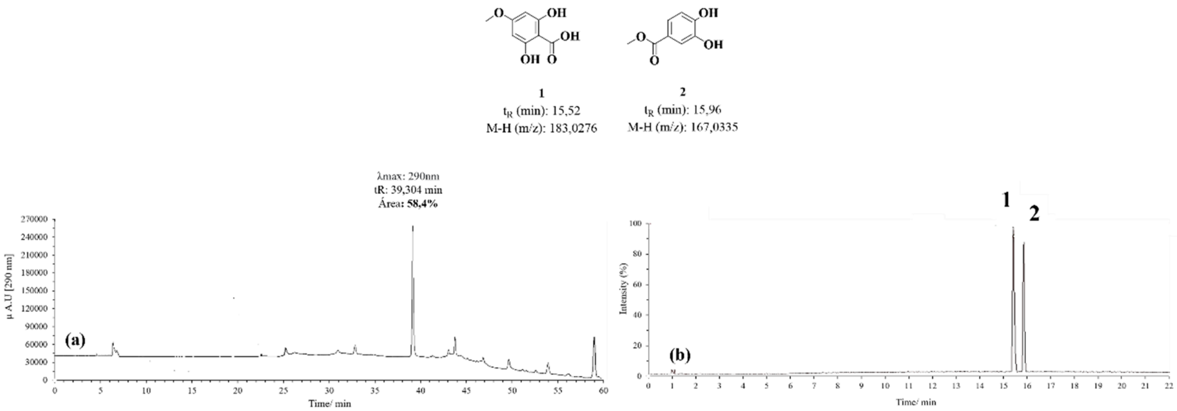

Figure S20. Chromatograms of rhamnetin MOx at pH 7 (D3): (a) HPLC-DAD, (b) UPLC-Q-TOF

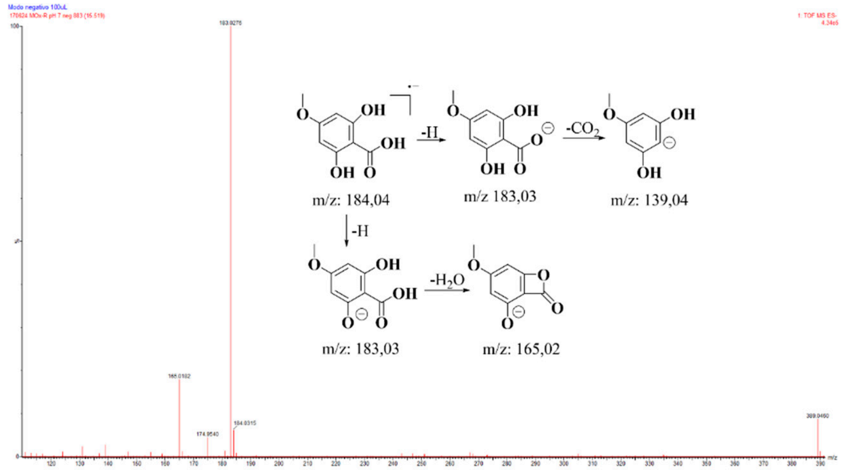

Figure S21. Mass spectrum of peak 1 from D3 MOx.

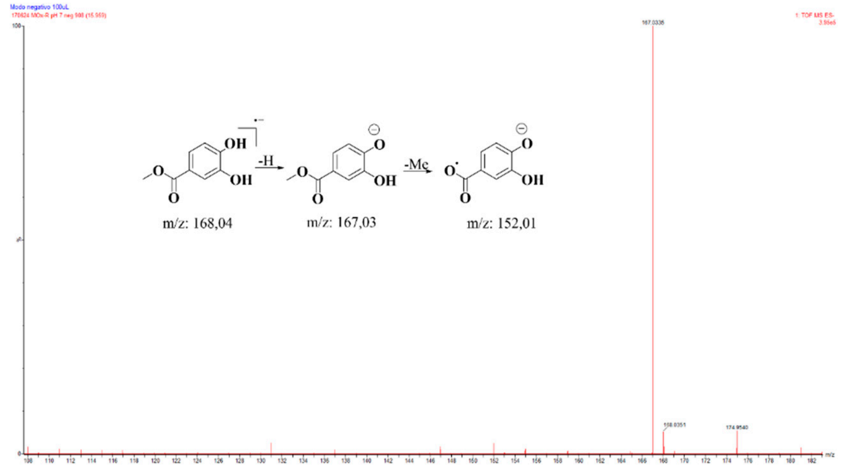

Figure S22. Mass spectrum of peak 2 from D3 MOx.

2. Biological Section

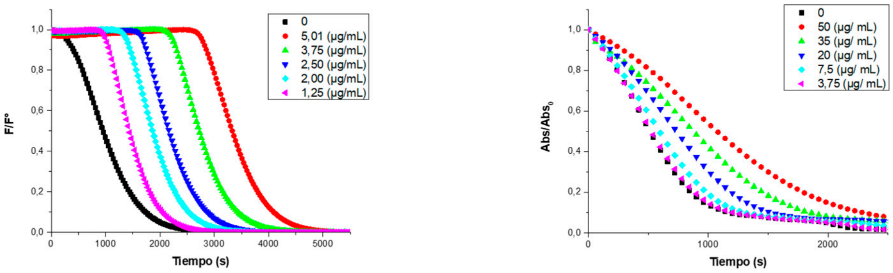

(a) **(b)**

Figure S23. ORAC graphs for Trolox: (a) ORAC-FL profile, (b) ORAC-PGR profile.

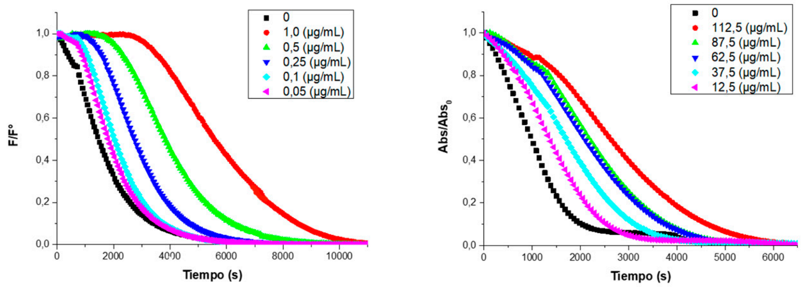

(a) **(b)**

Figure S24. ORAC graphs for quercetin MOx at pH 2: (a) ORAC-FL profile, (b) ORAC-PGR profile.

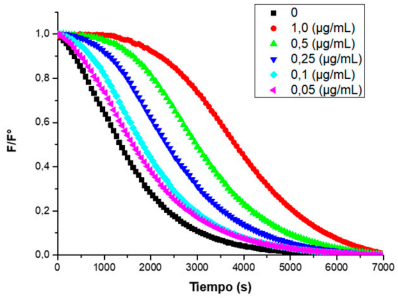

(a)

Figure S25. ORAC graph for quercetin MOx at pH 7: (a) ORAC-FL profile.

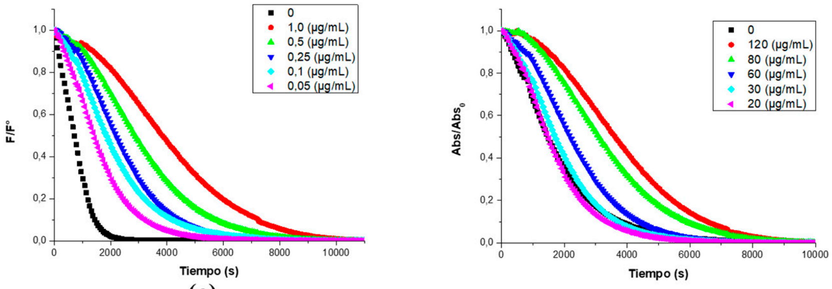

(a) **(b)**

Figure S26. ORAC plots of kaempferol MOx at pH 2: (a) ORAC-FL profile, (b) ORAC-PGR profile.

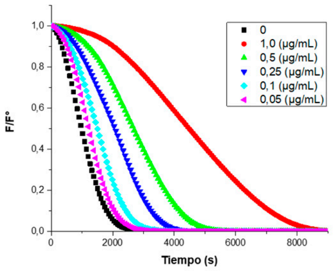

(a)

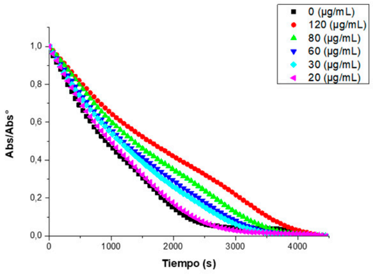

(b)

Figure S27. ORAC plots of kaempferol MOx at pH 7: (a) ORAC-FL profile, (b) ORAC-PGR profile.

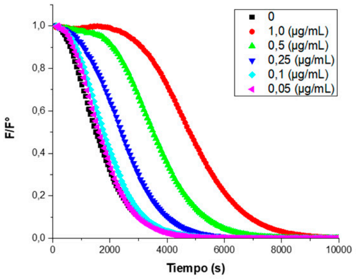

(a)

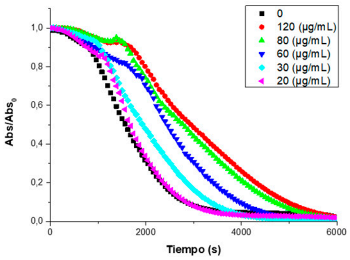

(b)

Figure S28. ORAC plots of rhamnetin MOx at pH 2: (a) ORAC-FL profile, (b) ORAC-PGR profile.

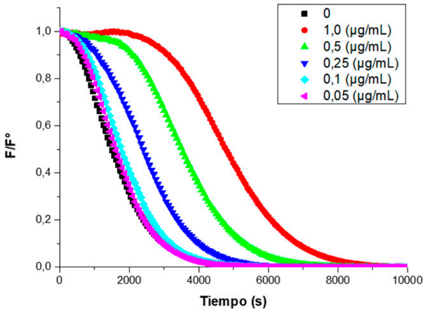

(a)

Figure S29. ORAC plot of rhamnetin MOx at pH 7: (a) ORAC-FL profile.

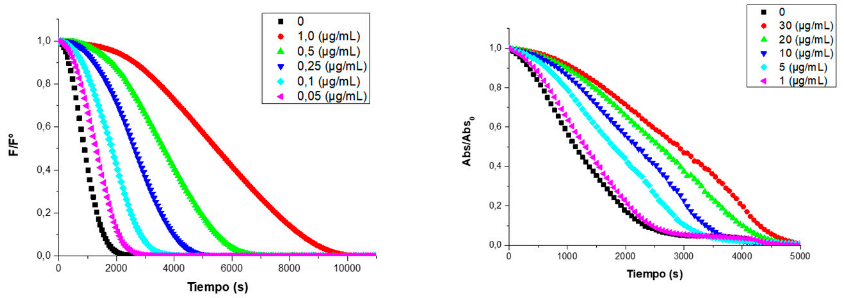

(a) **(b)**

Figure S30. ORAC plots of kaempferol: (a) ORAC-FL profile, (b) ORAC-PGR profile.

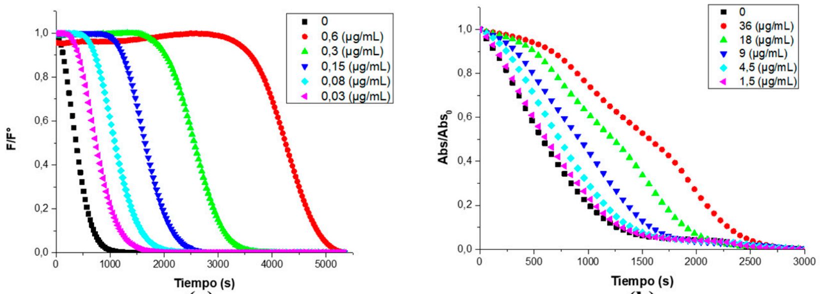

(a) **(b)**

Figure S31. ORAC plots of quercetin: (a) ORAC-FL profile, (b) ORAC-PGR profile.

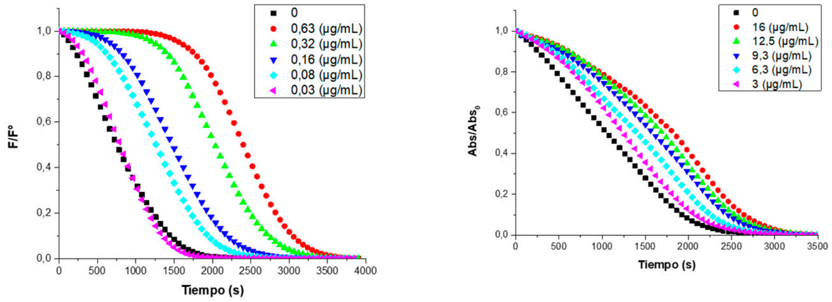

(a) **(b)**

Figure S32. ORAC plots of rhamnetin: (a) ORAC-FL profile, (b) ORAC-PGR profile.

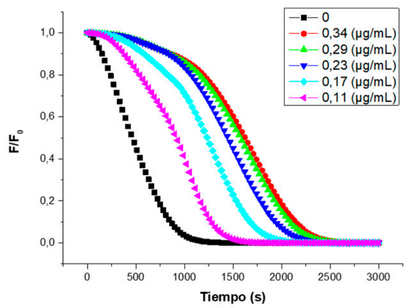

(a)

Figure S33. ORAC plots of luteolin: (a) ORAC-FL profile.

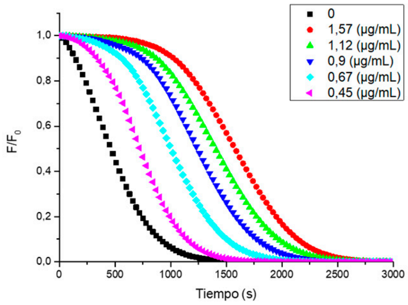

(a)

Figure S34. ORAC plots of ferulic acid: (a) ORAC-FL profile.

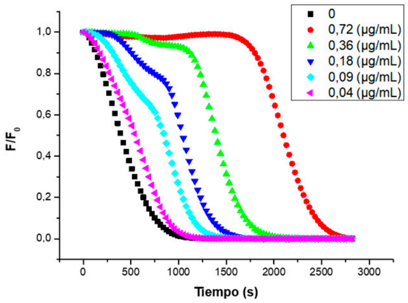

(a)

Figure S35. ORAC plots of rosmarinic acid: (a) ORAC-FL profile.

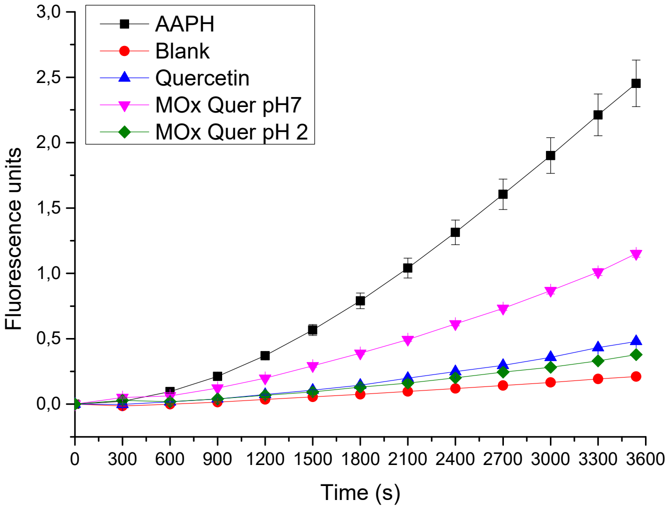

Figure S36. CCA plots for quercetin and MOx quercetin of pH2 y PH7.

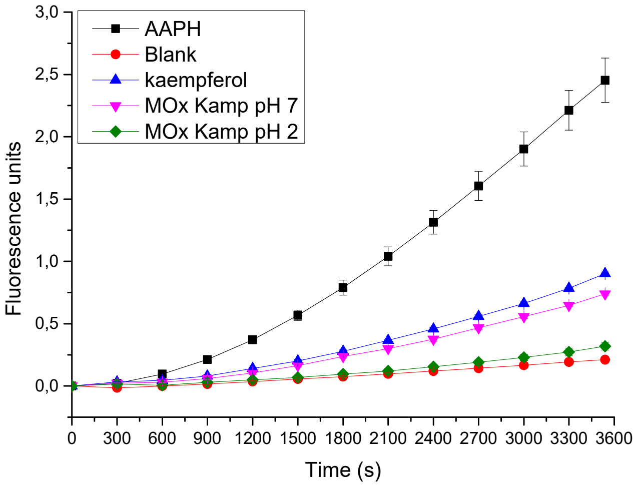

Figure S37. CCA plots for kaempferol and MOx kaempferol of pH2 y PH7.

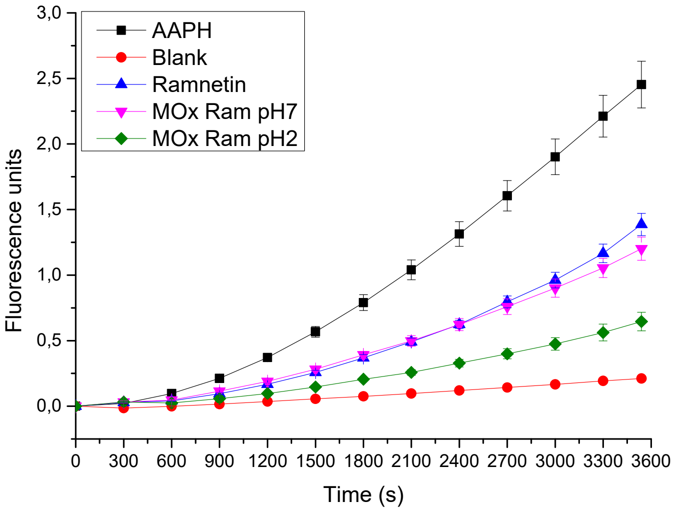

Figure S38. CCA plots for ramnetin and MOx ramnetin of pH2 y PH7.

3. Computational studies

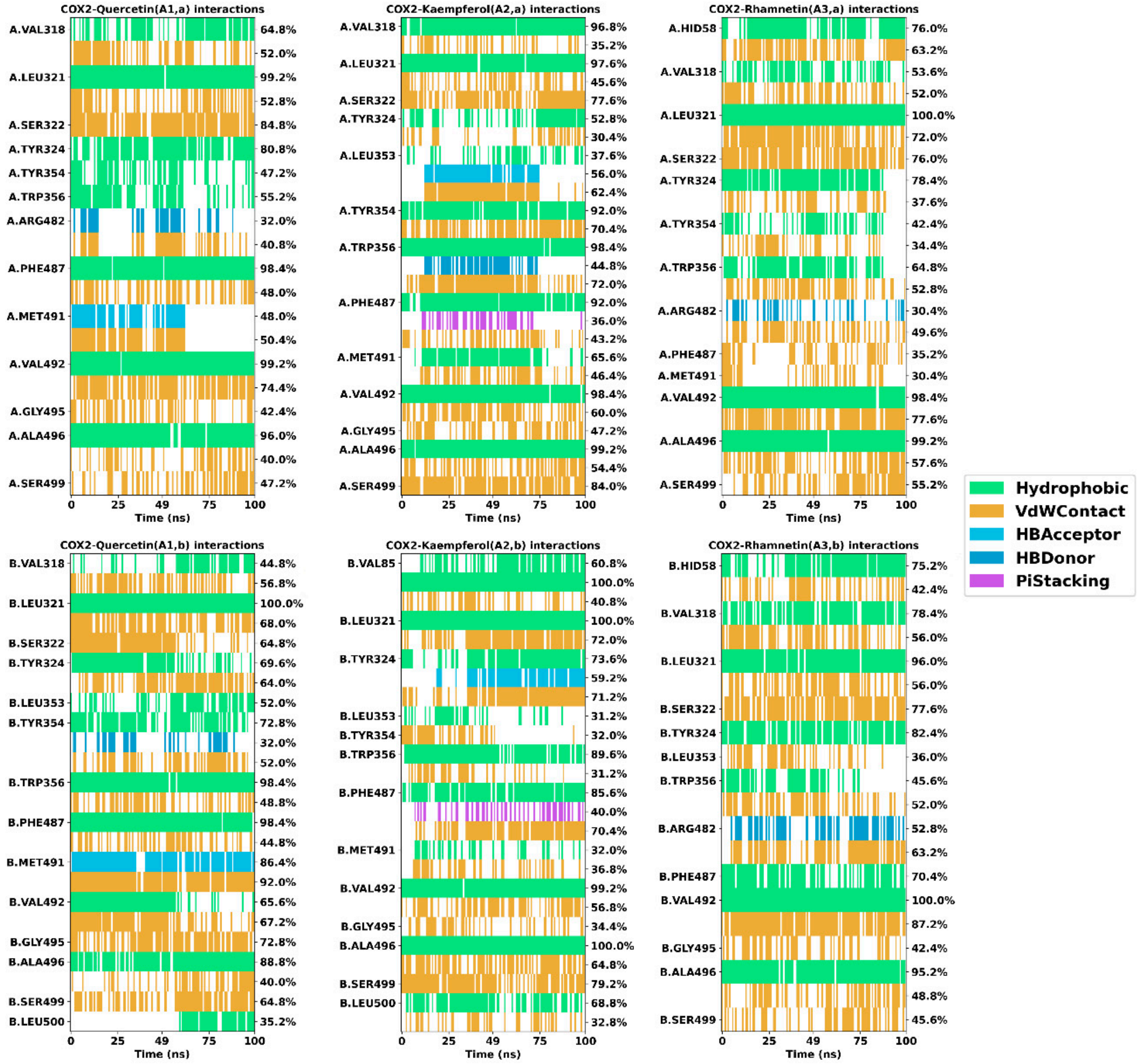

Figure S39. Interaction types between the COX-2/compounds complex (1).

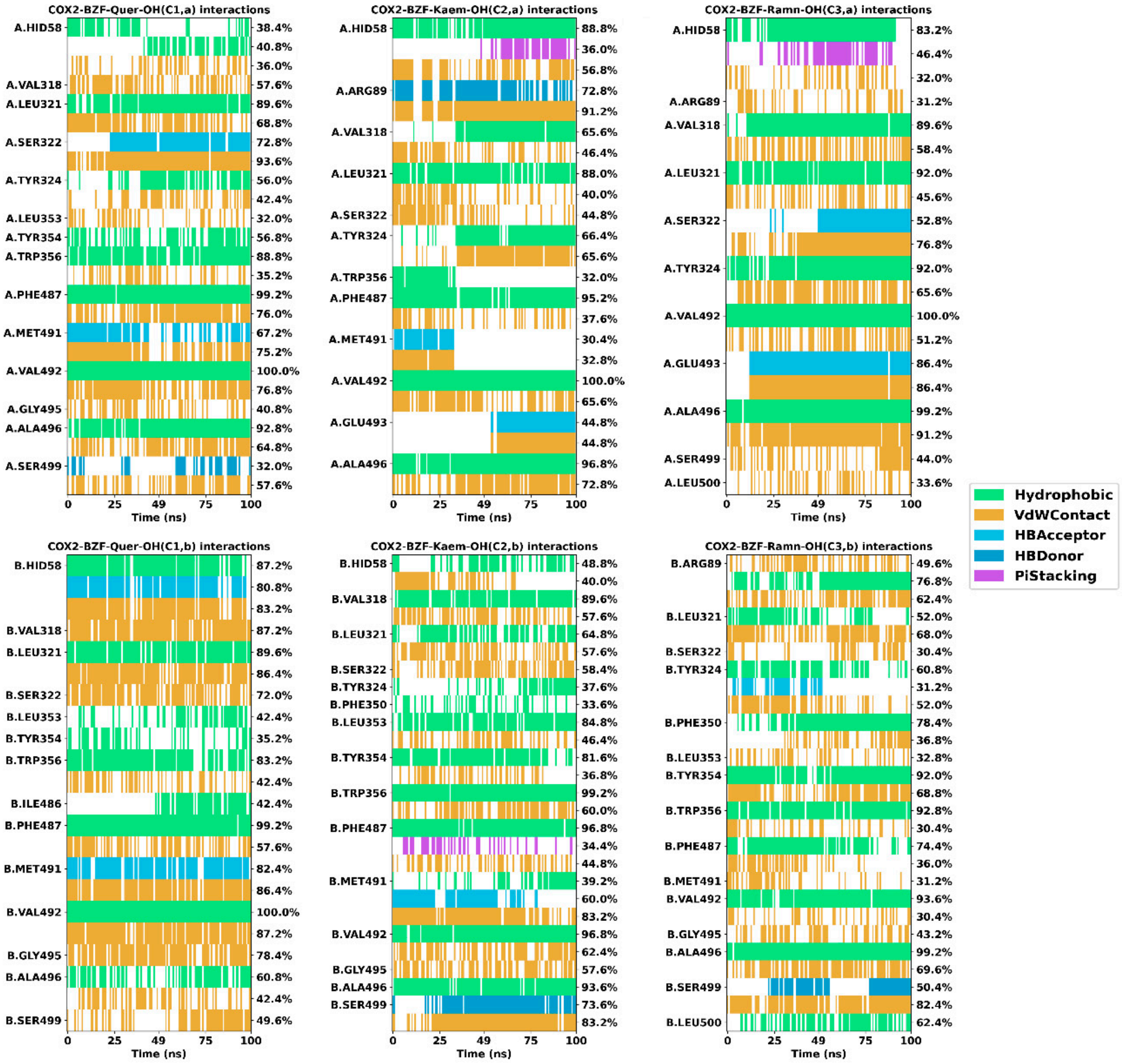

Figure S40. Interaction types between the COX-2/compounds complex (2).
